# Social bots spoil activist sentiment without eroding engagement

**DOI:** 10.1038/s41598-024-74032-0

**Published:** 2024-11-06

**Authors:** Linda Li, Orsolya Vásárhelyi, Balázs Vedres

**Affiliations:** 1 Department of Methodology, London School of Economics and Political Science, Columbia House, Aldwych, London, UK; 2 Oxford Internet Institute, University of Oxford, 1 St Giles, Oxford, UK; 3 Center for Collective Learning, Corvinus Institute for Advanced Studies, Corvinus University, Budapest, Hungary; 4 Institute of Data Analytics and Information Systems, Corvinus University, Budapest, Hungary; 5 Democracy Institute, Central European University, Budapest, Hungary; 6 Department of Network and Data Science, Central European University, Vienna, Austria

**Keywords:** Social bots, Human–bot interaction, Information cascades, Political communication, Protests, Psychology and behaviour, Computational science

## Abstract

Social bots are highly active on social media platforms, significantly affecting online discourse. We analyzed the dynamic nature of bot engagement related to Extinction Rebellion climate change protests in 2019. We found bots to impact human behavior more than the other way around during active discussions. To assess the causal impact of bot encounters, we compared communication histories of those who interacted with bots with matched users who did not. There is a consistent negative impact of bot encounters on subsequent sentiment. The impact on sentiment is conditional on the user’s original support level, with a more negative impact on those with a favourable or neutral stance towards climate activism. Political ’astroturfing’ bots induce an increase in human communications, while encounters with other bots result in a decrease. Bot encounters do not change activists’ engagement levels in climate activism. Despite the seemingly minor impact of individual bot encounters, the cumulative effect is profound due to the large volume of bot communication. Our findings underscore the importance of monitoring the influence of social bots, as with new technological advancements distinguishing between bots and humans becomes ever more challenging.

## Introduction

Social media has become the primary channel to engage in political discussions, and to participate in collective action over the past decade^1–3^. However, on social media there are automated agents as well, among them “social bots”, that are increasingly active in our social and political publics^4^, creating content and interacting with humans with ever increasing sophistication^5^. This hybrid ecosystem where algorithmic agents and humans co-exist can fundamentally alter the nature of democracy, political accountability, transparency, and civic participation^6^. Automated accounts can propagate a large volume of messages at minimal expense, and can engage with users with a fast reaction speed^7^. As a consequence, the algorithmic share of social media communications is now on par with human participation: Automated users were estimated to be responsible for generating 10–40% of tweets in recent political events, such as the 2016 US presidential election, the Brexit referendum, the yellow vests movement, the Catalan referendum, or the 2019 United Nations Climate Change Conference^8–10^.

Social bots—especially those intending to mimic human behavior—can disrupt online political discussions^11^, and significantly influence political debates and activism^11–13^. Such bots are frequently designed to pass as human accounts, and occasionally also mimic known political figures and government accounts to gain the attention and trust of human users^14^. Bots are often highly active during the flare-up of discussions around new political events^15^, and disseminate targeted messages ranging from fabricated news to contentious, divisive, and negative content^16–18^, blending legitimate messages and misinformation. Furthermore, bots are also often deployed in orchestrated efforts to generate the facade of a seemingly vibrant discussion conforming with hidden agendas^19,20^ by retweeting each other (known as “astroturfing”), targeting susceptible users^8^.

Although social bot presence has been studied before at the macro scale, less is known about the micro-level impact of human-bot encounters on subsequent human activism. Research on bot-human interaction found that bots can often hijack the topics and overall tone of human discussions^10,16^, and they often increase the visibility of extreme views^8,16,21^, influence sentiment around topics^22^, and intervene in human communication flows^23,24^. Small-scale simulations and experiments indicate that bots can alter expressed human values^24^ and behaviors^25^, particularly driving users towards more extreme viewpoints^26^, and potentially impact human’s level of online activity^27^ as well as offline political participation^28^. However, there is little empirical evidence regarding the capacity of bots to modify human behavior in real-life political communication^17,19,23,29^.

Today, online activism constitutes an essential part of democracy. Online activism can be defined as the strategic use of digital communication technologies by individuals or groups to engage in political and social change efforts. These efforts include social media campaigns, online petitions, digital protests, and the diffusion of messages through digital platforms to mobilize individuals or communities around specific causes, and advocate for policy changes^30^. However, most research on social bots focuses on automated interventions in institutionalized political processes such as elections^21,22,31,32^, while few studies examined the role of bots within activism related to online protests^10,23,33^. The dynamics of social media use in online activism differs markedly from communication around other political events^34–37^. Since social bots are designed to be more responsive than humans^38^, it is common to observe an increase in bot activity during the peak of heated online debates, followed by a decrease in their presence^8^. While the bursty nature of social media communication of protests is well known^39,40^, the impact of social bots on human activity during and after bursty periods has not been investigated.

How does interacting with social bots impact human behavior in online activism? We address this question with data on X (Twitter) discourse on climate-change-related social movements. We analyze the dynamics of bots and humans engaging with each other, and we also compare the difference in impact of direct communication with bots to activists who did not directly interacted with bots. We focus on events of direct communication with bots—when humans engage with bot messages in writing (replying, mentioning or commenting bot tweets)—and not merely events of seeing bot communications, as bot messaging today is highly common, and seeing one automated message should not have much impact. Our analysis focused on protest-related discourse during a series of protest events that erupted from November to December 2019. We decided to concentrate on online activism related to climate change as our case study, as algorithmic threats to engagement in climate change activism can have profound consequences on societal agreement on the public good in a critical issue^41^. We analyzed communication around the Extinction Rebellion (XR), as the highest profile activist group online.

The topic of climate change has been shown to attract highly engaged, active, and committed participants^42^, while there is also substantial bot activity^43,44^. This enables us to analyze the impact of human-bot interactions on humans during information cascades^19^, and measure the effect of bot-human interactions on tweeting activity^27^ and sentiment^26^. Our findings contribute to the growing body of research on machine behaviour^6^, particularly in terms of understanding how rapidly developing hybrid human-machine systems could potentially modify human opinion over an extended period.

Early work related to human–bot interactions on social media found that user sociability and network size predict who will be interacted by a bot^15,45^. Our results show that bots have become so widespread that it is unlikely that an active user of X will *not* meet a bot online. Our research extends previous work by focusing on quantifying the impact of direct human-bot communication. We found that bot type matters for the impact on users’ tweeting activity, and the initial level of support a user has toward the climate change movement determines how a bot encounter impacts their sentiment on climate change. Our results have important policy implications for increasing platform transparency in how they handle automated profiles.

## Results

### Proportions of bot and human communication

We found $48\%$ of all accounts to fall into the bot category within our sample from Twitter (44, 121 of the total 93, 499 users). We identified automation through a combined approach^46,47^ that integrates the results of commonly used bot identification methods, Botometer^48^ and our self-trained machine learning-based algorithms. Botometer is a widely adopted open source tool to identify bots on Twitter^49^. Our self-trained models used various data sets tailored to identify users with automated behavior that attempts to mimic humans on social networks, especially in the context of political behavior^48^. Bots reported in the main text are the combined results of these two models, with a fixed threshold for the Botometer ($CAP>=0.65$). Since the concept of “bot” encompasses varying degrees of automation, using one fixed bot classification threshold is always a simplification. Therefore, we repeated all analyzes reported in the main text at various bot thresholds, and we report these results in the SI. (See 5, 5.2 and SI Bot Identification for more details. Supplementary Information (SI) Table S3 shows details of the training set and data used for bot identification, while Figure S1–S4 shows metrics used for fine-tuning bot detection models).

**Fig. 1:**
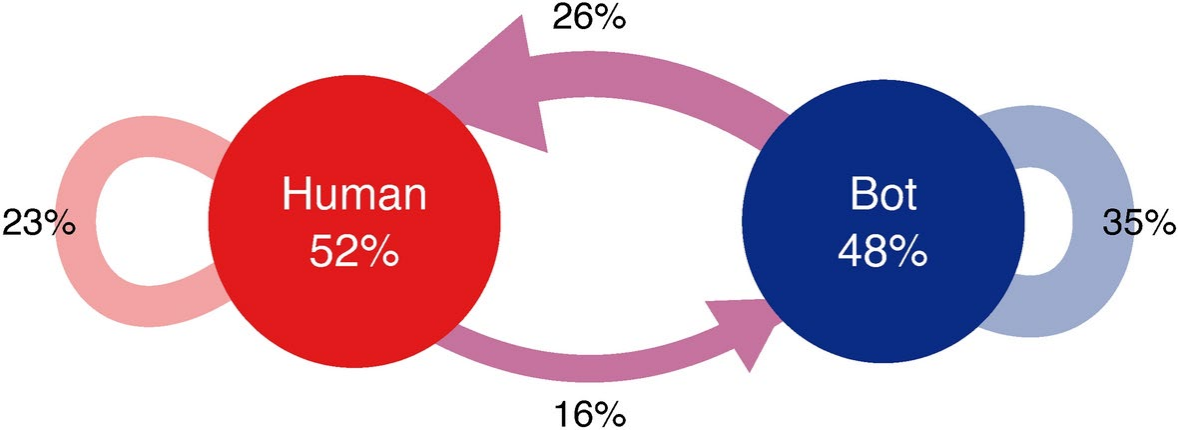
Information flow of humans and bots. The directions and amount of tweets retweeted between bots and humans.

Figure 1 shows the information flow between bots and human users. 81% of tweets were replies or retweets in our database, and 51% of these retweets originated from bots. In general, bot activity is mainly the posting of original messages or the retweeting of each other (35% of all retweets and 71% of bot retweets were retweeting other bots), and only a small portion of bot retweets were retweets originating from humans (29%). At the same time, humans spread roughly the same number of messages produced by bots (54%) and humans (46%). If the bot threshold is increased to $CAP>=0.75$, still 48% of the retweets originated from bots, and 45% of the human retweets originated from bots (see SI Information Flow and SI Tables S6 to S8 for information flows).

### Temporal co-dependence of bot and human tweeting

We found that the quantity, intensity, direction and the sentiment of the information flow between humans and bots are highly topic-dependent. We identified seven topics related to XR protests associated with news events, political campaigns, or outbursts of sentiments (for example, climate change denial) by applying bi-term topic models^50^. (See 5, 5.3, Topic Modeling for more details and SI Table S5 for a brief summary of the topics, including their content, top hashtags, and sample tweets). Out of these seven topics, four were highly bursty: Topic-related activity increased sharply periodically and then decreased suddenly^51^, resulting in information cascades. Information cascades occur when users follow the behavior of other individuals on social networks (e.g. retweeting the same message)^52^. (See Table S9 in SI Burstiness Scores).

**Fig. 2:**
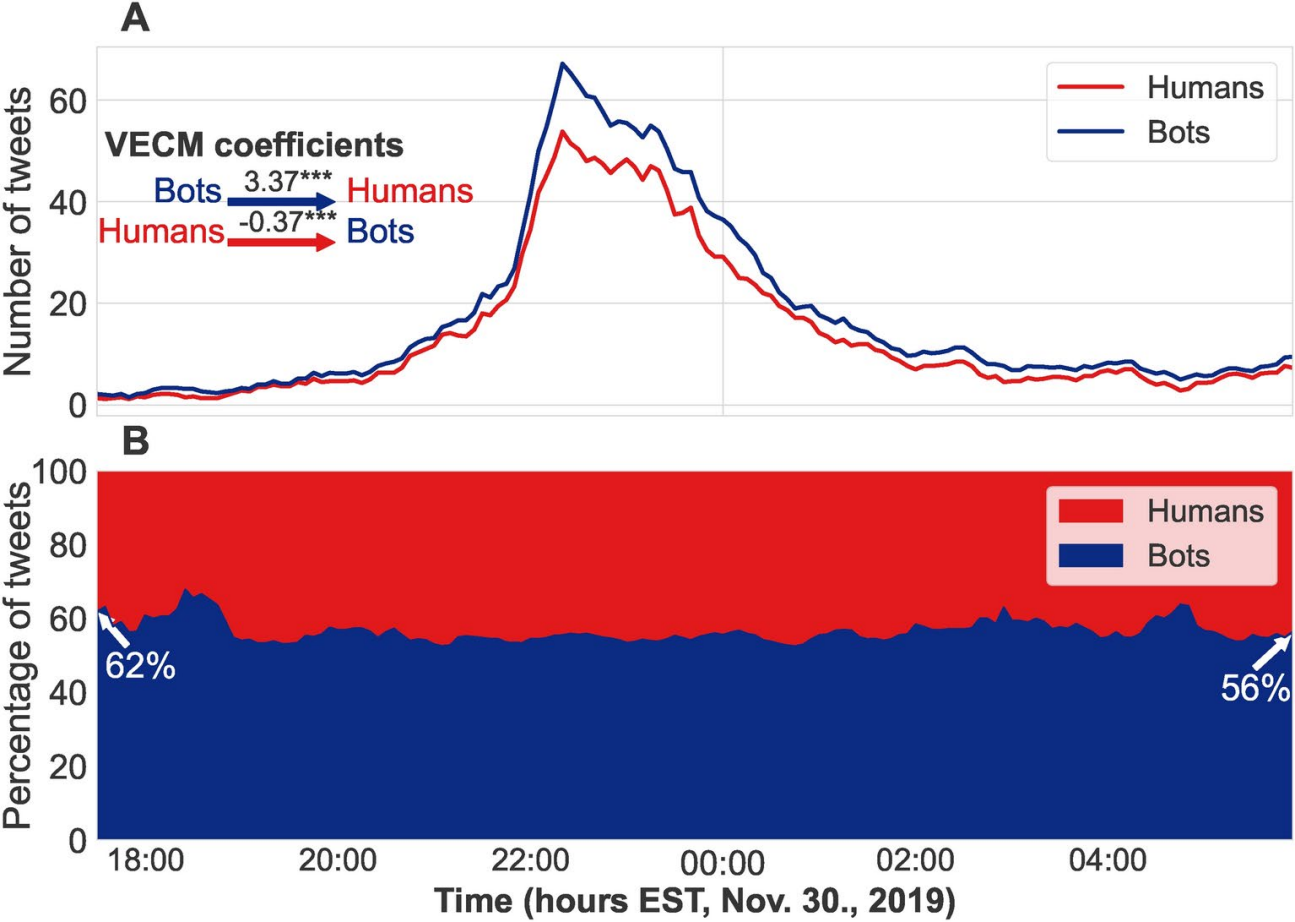
Panel (**A**): Number of tweets posted by bots (blue line) and humans (red line) in a cascade mutually-driven by bots and humans within the “Anti-XR protest” topic. Panel (**B**): Ratio of bot and human generated tweets throughout the time period of the cascade. The number of tweets is the rolling average aggregated on 5-minute intervals. Bots are classified as users who has a overall bot probability no less than 0.65.

Figure 2 shows the temporal distribution of human and bot tweets aggregated at a 5-minute interval within an illustrative bursty period of climate change discussions on Twitter. Although human users (red line) generated a higher volume of tweets during the cascade’s peak, the tweeting frequency trend is influenced by bot. Table 1 shows the results of Vector Error Correction Models (VECMs). VECM is a statistical model used to analyze and estimate whether one time series could be used to predict another after introducing a time lag and potential confounds^53^.

We found that in 3 out of 4 identified cascades, the number of bot tweets during bursty periods could be used to predict human activity, and the sentiment of tweets by humans could predict the sentiment of bots’ tweets. Bots impacted human tweeting activity significantly stronger in the case of “Disruptive engagement” (Wald = 10.06, *p* = 0.0015) and “Anti-XR protests” (Wald = 45.36, *p* = 0.000), but tweeting activity related to “Politicized Activism” was mutually driven by both bots and humans. (Wald = 0.76, *p* = 0.39). Humans impacted bots’ sentiment significantly more negatively in the case of “Anti-XR protests” (Wald = 3.66, *p* = 0.048). In the case of “Politicized Activism”, sentiment was also mutually driven by both bots and humans, with no significant difference in coefficients strength (Wald = 1.65, *p* = 0.198). The effect remains consistent across bot CAP thresholds ranging from 0.50 to 0.75 for 3 of the 4 cascades identified. (See more details on Burstiness in Data and Methods, Identifying cascades, SI Cascades, SI Tables S9–S20 for Results across different CAP thresholds, different time lags, for full time period of our analysis instead of cascades, and for Topic Burstiness Scores).

**Table 1:** Vector error correction models (VECM) testing the relationship between the amount and sentiment of bot and human communication with 20 minutes time lags.

Topic	Amount bots$\rightarrow$humans	Amount humans$\rightarrow$bots	Wald test	Sentiment bots$\rightarrow$humans	Sentiment humans$\rightarrow$bots	Wald test
“Football game protests”	$-$0.14	0.06	1.72	$-$0.01	0.13	2.05
“Disruptive engagement”	57.89***	0.01	10.06**	$-$0.04	0.34**	2.70
“Anti-XR protests”	3.37***	$-$0.38***	45.36***	$-$0.08	$-$0.65***	3.66*
“Politicized activism”	0.54**	0.17***	0.76	5.09***	4.12***	1.65

$$^{*}\textit{p}< 0.05; ^{**}\textit{p}< 0.01; ^{***}\textit{p} < 0.001$$. VECM-s were used to determine whether bot activity predicts human activity (Bots to Humans) or human activity predicts bot’s (Humans to Bots) in the identified four cascades. Wald tests were performed to test whether coefficients of Bots to Humans and Humans to Bots predictions are significantly different. Modelling were based on 30-minute time lags.

### Predicting the impact of direct bot interactions

To test whether direct communication with bots (replying, mentioning or commenting bot tweets) has any influence on human users—beyond their above shown immediate collective effect in bursty periods—we developed two models focusing on (1) Amount and (2) Sentiment. Specifically, we analyzed how human communication evolves over a span of 30 days after the first direct interactions with a bot in the discussion related to climate change on Twitter. The first model captures the inclination to “speak out” quantified by the average number of tweets posted related to the XR protest. The second model predicts the change in sentiment about the climate change protest (measured on a scale ranging from $-1$ to 1, with $-1$ representing the most negative sentiment and 1 the most extreme sentiment).

**Fig. 3:**
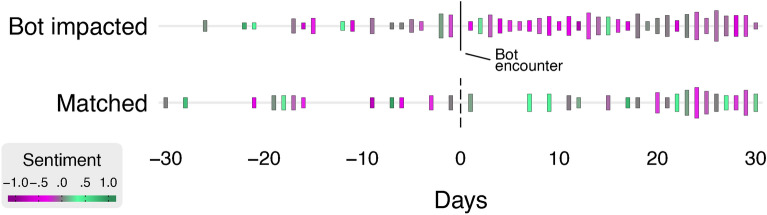
Two examples of Twitter timelines. Top timeline shows daily tweet counts and daily mean sentiment for a human account that directly interacted with a bot on day 0; bottom timeline shows a matched account without a direct bot interaction. Bar heights are proportional to tweet counts, color indicates sentiment from $-1$ to 1.

To quantify the impact of direct bot interaction on human communication change, we used a three-step process. This includes: (1) we sampled $N=303$ users who directly interacted with bot accounts (bot-exposed)—replied or commented to a tweet/comment originated from a bot; (2) we collected a matched sample of $N=184$ users, who were active in the XR related protest discussion on Twitter but had no direct interaction with bots. Matching users were selected on the basis of the similarity score calculated pairwise to our original sample considering publicly available metrics on human users’ profiles. (See Data and Methods, Matching sample for more details.) (3) Figure 3 shows two example timelines, one human user who directly interacted with a bot (the interaction is shown at time 0), and a matched user that did not encounter a bot.

(3) Finally, we applied Difference-in-Difference (DiD)^54^ regression models to quantify the casual effect of bot interaction on outcomes by comparing a set of humans who directly interacted with bots (human replying or commenting bot tweets) with those who did not. Our observation units are the daily activity of human users relative to the time of interaction with bots - 30 days *before* and 30 days *after*. This setting allows us to quantify the prolonged impact of bot interaction on the frequency of tweeting and sentiment of tweets compared to users who did not meet a bot.

**Fig. 4:**
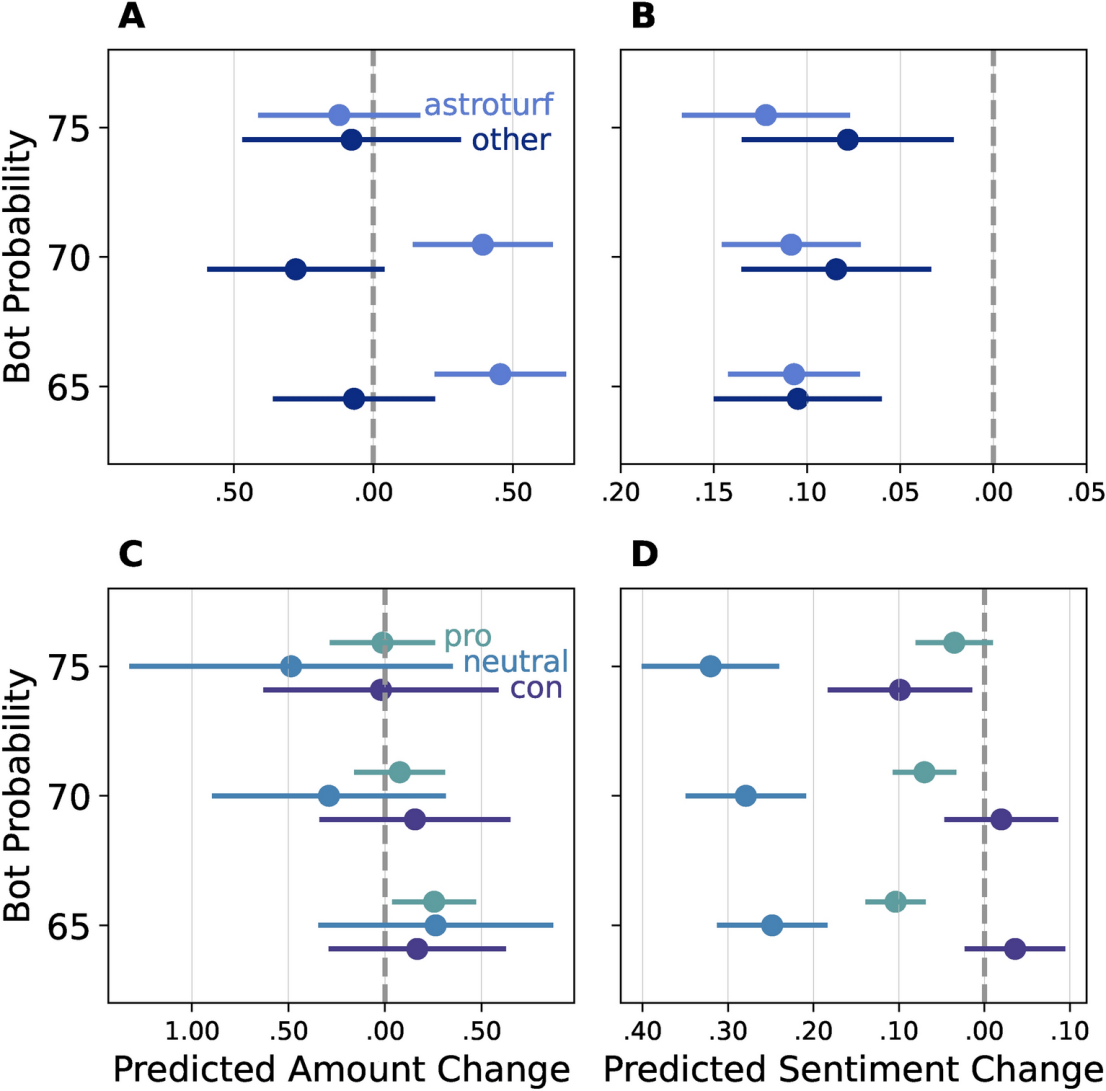
Predicted change resulting from a bot interaction. Panel (**A**,** C**): Predicted change in Amount (number of tweets). Panel (**B**, **D**): Predicted change in Sentiment. Panel (**A**,** B**) separate predictions (by color) are shown for astroturf bots (light blue), and other bots (dark blue), while Panel (**C**,** D**) separate predictions by users support level towards XR—supporters (green), neutral users (blue), anti-XR (purple).

Botometer provides prediction values for belonging to seven subbot categories ranging from 0 to 5. We classify a bot into a subcategory if its bot-specific probability is greater than 2.5. Our biggest group is ‘miscellaneous other bots’ which are bots that are similar to various type of manually annotated bots (23%), followed by manually labeled political bots, so-called ‘astroturfs’ (14%),‘fake followers’ bots purchased to increase follower counts (7%), ‘self declared’ bots from botwiki.org (5%), ‘spammers’ (2%) and ‘financial bots’ (0.4%). Although ‘miscellaneous other bots’ are quite well represented within the XR discourse online, only 17% of human users had direct interaction with them. As implied by the topic of our analysis, the individuals in our sample primarily engaged with ‘astroturfs’ (38%). These automated accounts are specifically designed to participate in political discussions^2^, leading us to analyze users interacting with astroturfs separately from those who interacted with non-astroturfs.

Figure 4 visualizes the predicted daily change in the average number of daily tweet counts (Panel A), and the sentiment of the tweets (Panel B) grouped by different bot probabilities (65,70,75) and subbot category (astroturf or other type of bots). We found that interaction with astroturf bots results in an increase in the number of tweets, while communication with other kind of bots result in a decrease in the number of tweets. This suggests that the most politically relevant bot category—astroturf bots—drive the conversation by provoking engagement from human users, while other kinds of bots have a rather negative, silencing effect. Regardless of the bot type, direct interaction with a bot decreases the average sentiment of human users (See Tables S22–S27 for DID models).

Astroturfs tend to be mobilized in a targeted way against users with a specific opinion^15,55^. Therefore, we classified bot-exposed and matched human users by their support of XR Protests: Supporters (Pro—52%), Neutral (27%), and Anti-XR (Con—21%) using ChatGPT. (See Materials and Methods on Support Categorization). Figure 4, panel C, D indicates that human users who support or have a neutral opinion about XR are significantly affected by interacting with bots, while anti-XR users are not affected. Bot interaction has the strongest negative impact on the sentiment of bot-exposed users with neutral opinion, indicating that bots might target those users whose opinion can be changed^15,56^. The change in the number of tweets was significantly increased by bot interaction for XR supporters within the least selective bot probability category (65), although the trends are similar in the more selective categories (.70, .75). In these models, we control for the astroturf score of the interacted bot, which has a strong positive significant relationship with the change in amount. Our results indicate that the type of bot matters more for the activity change than the original support towards protests. However, the level of sentiment change depends on the original support level of users exposed to bots (See SI Table S28–S33 for DID models).

We also investigated whether bot-affected users alter their levels of support as a result of bot interaction. There were no significant differences between the distribution of bot-exposed users’ support level before, and after interacting with a bot (Mann-Whitney U = 45536.00, *p* = 0.86).There was no significant difference either between users exposed to bots and those matched in terms of the change in support level.(See Model Tables S34–S35 in SI on Opinion Change). Out of the users who interacted with bots, only 9% experienced a shift towards neutrality, while 10% of the opinions of the users in the matched group became more neutral towards XR protests. Additionally, 7% of the users exposed to bots and 6% of the matched users changed their opinion completely, shifting from negative to positive or the other way around.

The effect of bot interaction on human behaviors remains statistically significant after controlling demographic variables (location of users), for retweets to news media reports, and with a sample matched with different matching methods (See Model Tables S36–S39 in SI on other robustness checks).

## Limitations

There are four potential limitations to our current research design. The first is the relatively short time range and sample size of our dataset on XR protests. Our dataset covers several waves of XR protests within a month, but XR is a global phenomenon that has been going on for several years. However, comparing our sample size to previous studies on online activism and political communications on Twitter^34,36^, we believe that it is a valid and representative case to illustrate the studied aspects of bot activities on online activism. The Twitter academic product track API (available at the time of data collection) provided the full archive of tweets based on specific search queries; therefore, our dataset is a comprehensive sample of the online record of bot and human activities during the protest period.

The second limitation concerns our binary bot detection method. It has three main drawbacks, each of which we address through additional efforts and measures. First, similar to other bot identification approaches^10,48,57,58^, and due to the nature of unsupervised learning, our method cannot be 100% sure whether a user classified as a bot is genuinely a bot. Second, the concept of “bot” encompasses varying degrees of automation among Twitter users, and using a fixed bot classification threshold overlooks these nuances. We are aware that being a bot, similar to the concept of gender^59^, is not a binary classification problem. Many human users apply automation to increase their efficiency^60^, which does not turn them into bots, but they are no longer non-automated human users. Third, a fixed threshold-based approach can still lead to false positives and false negatives, potentially undermining the validity of our causal inferences regarding bot activities. To address those concerns, we ran our models with varied CAP scores. We also performed a series of robustness checks involving various thresholds for all of our analysis and reported them in the main text. (See Discussions, SI Information flow, Cascades, and Difference-in-Difference regression results).

The third category of limitations is due to the automatized methods used to label users’ support towards XR protests and sentiment of the collected tweets. We are aware that these methods are not perfect^61^. Therefore, we validated these results by two manual coders based on a subset of tweets and users timelines. We found that ChatGPT produced support level values correlated highly with human coders ($corr=0.88$). Based on assessments of the accuracy of the VADER algorithm used for sentiment analysis, we found that it categorizes slightly more tweets as neutrals than human coders did. However, we found that the results are sufficiently accurate in all sentiment categories. (See Precision, Recall and F-score by sentiment category in SI, Table S21)

The final limitation concerns our Difference-in-Difference sampling design. We collected a comprehensive archive of tweets during the sampling period to ensure that our control (matched) group did not interact with social bots during that time. However, we cannot definitively confirm whether matched users had interacted with bots prior to the sampling period. Additionally, while we accounted for several confounders, unmeasured factors may still influence the observed differences between the treatment and control groups. Such limitation introduces a potential issue for our causal inference, and necessitate careful interpretation of the relationships.

However, our findings show that orchestrated bot activity was highly concentrated and bursty around key protest-related events, suggesting that bot influence was reactive to these news peaks. It is unlikely there was much sustained bot activity before the outburst of protest events, which implies that any potential effects from earlier interactions would be minimal. Furthermore, based on our observation of changes in sentiment and retweeting behavior, the effects of bot interaction appear to be short-lived. As a result, any bot interaction that occurred prior to our sampling period would likely have diminished by the time our analysis begins. Therefore, it is unlikely that pre-sampling bot interactions would significantly impact our estimates.

Furthermore, our analysis already reveals significant differences in behavior between users who interacted with bots and those who did not during the sampling period. If some control group users had interacted with bots before the sampling period,we would expect them to display similar behavioral changes, like shifts in sentiment. In this case, both groups would have users who have experienced bot-induced behavior changes. As a result, the observed differences between the two groups would become smaller than the actual effect, making our estimates of the treatment effect more conservative.

## Discussion

Bot presence is considerable and possibly increasing in the public sphere. Even with stricter thresholds, bot activity is higher in our sample than in previous related work published on the 2017 Catalan referendum by Stella et al.^16^, where only 19% of all interactions were from bots to humans. It was shown that tweets from conservative bots are more retweeted by humans, which can indicate that this difference is not only due to the continuously increasing presence of bots^62^, but could be due to the highly politicized and international nature of our context.

Even though we considered only one public in detail, we replicated our descriptive analysis of information flows on similar data from Twitter about #BlackLivesMatter movement. At the active phase of exchanges from the time of the first protest followed George Floyd death, we found that the patterns of how bots impact human communication are are not significantly different in the two context (See SI Figure S1 for more details). Although XR is one of the most prominent fields of protests online, with highly committed activists which can lead to increased bot presence and more targeted actions against them, the similarity between the two cases indicate that increased bot presence is a general threat that activists face in social media.

We have adopted a dynamic approach to political communication and have found that bots should not be thought of a constant presence, but rather bots are driving the amount of heated and bursty discussions, and react in a dynamic fashion to human sentiment. Our temporal analysis of human and bot activity showed that in 2 out of 4 identified cascades bots impacted human tweeting activity significantly more, however humans drove the sentiment of the communication. We also found that bots and humans can mutually drive cascades, depending on the topics and the intensity of the debate.

To quantify the causal impact of human–bot communication, we have compared the communication histories of human users who have directly communicated with a bot with those matched human users who have not. This allowed us to see a consistently negative impact of any kind of bot interaction on the sentiment of subsequent human communications: Humans who have interacted with a bot displayed considerably more negative sentiment than matched users.

The change in tweeting activity after a bot interaction depends on the nature of the bot: On one hand, decidedly political astroturfing bots (aiming to influence public opinion behind an impression of grassroots opinion) result in an increased activity. On the other hand, interacting with other kinds of bots (spammers, fake followers) results in a decrease in activity. However, change in sentiment towards the protests depends on the original support level of the user, supporting, neutral, or against XR. Our results indicate that bots might target users whose opinions are easier to change, since the sentiment of bot-exposed users with neutral opinion decreased the most. However, bots do not make human users switch their support level. In sum, bot interaction is not without impact, even if one encounter itself has only a small effect (it takes about two bot encounters to induce one additional tweet). Nevertheless, since there is an exceeding amount of bot communication, these small impacts add up to influence the public sphere in a profound way.

Although our analysis covers a period of time prior to the launch of ChatGPT (2022.11.30), it is becoming increasingly difficult to identify bots due to the rapid advancement mimicking human behavior^63^. As large language models are becoming widespread and easily accessible through APIs, new social bots can act extremely human, making it currently almost impossible to distinguish between bots and humans, even for experts^64,65^.

Therefore, it is crucial to have unrestricted access to social media data to assess the influence and prevalence of these new types of social bots, although recent trends show that social media platforms are less willing to share free data for research purposes^66,67^. Since financial evaluations are highly correlated with the size of the (human) user base^68^, platforms have no interest in quantifying the ratio of non-human accounts and their impact on misinformation^15^. Most users still underestimate the effect of bots on themselves, but as they are exposed to increased bot presence, they tend to prefer stricter bot-regulation policies^69^. That is why we welcome the news that the European Union requires larger platforms to provide researchers with access to data to study systemic risks arising from the use of their services, such as disinformation^70^. Such legislative actions can help the scientific community continue its work to understand the consequences of this abrupt change in technology that will alter the nature of human–bot interactions^64,71–73^.

## Data and methods

### Data

Our data is made up of Twitter activity around several waves of Extinction Rebellion climate change protests from 18 November 2019 to 10 December 2019. The dataset was collected from November to December 2022 via the Academic Research product track API provided by Twitter^74^, which enabled users to collect a full archive sample of historical tweets filtered based on keywords and conditions. We collected all tweets posted during this period of time that contained the keyword ‘Extinction Rebellion’, ‘climate change protest’, ‘XRebellion’, ‘XR’ and multiple variants of keywords with slightly different spelling. (The complete list of keywords used can be found in SI Table S2). In total, the final data set contained 201,010 tweets and 122,130 users.

### Bot identification

To identify social bots on Twitter, we used a combination of two sets of bot identification methods. The first is a popular Twitter bot identification tool known as the ‘botometer’ (formerly BotorNot), which was primarly used as a benchmark to compare other methods. The second is a set of our self-trained bot identification model trained with open source data of bots and humans to train supervised machine learning models.

Botometer is a publicly available tool that relies on machine learning. It is designed to calculate a score where low scores indicate likely human accounts, while high scores suggest likely bot accounts^75^. The algorithm considers more than 1000 features related to user profiles, friends, network structure, and activity patterns, among others. Another part of our bot identification pipeline comes from self-trained models. Training sets were derived from existing open-source data from Twitter accounts identified as ‘bots’ and ‘humans’.

We trained bot identification models with 70% training and 30% of testing set with five types of algorithms: random forest(RF), support vector machine(SVM), logistic regression(LOG), XGboost classification (XGB) and deep learning (DL). We developed two versions of our models with ten and twenty features that were proved to be most effective for bot identification by previous studies^10,76^. The evaluation of the models demonstrated that the RF, DL, and XGB models with 20 traits surpassed other models in terms of sensitivity (true positive rate), balanced precision, precision, and F1 score. Additionally, these models exhibited strong performance in an independent test carried out on a dataset consisting of influential bots and human mimics that were active during the 2018 midterm election of the United States.

In our final bot identification approach, we combined the results derived from both sets of bot identification methods. Due to the potential false positive issues inherent in both methods, they yielded somewhat divergent results^49^. To reconcile this disparity, we classified users based on the overlapping results of botometer *and* our proprietary algorithms (DL, RF, or XGB). If both the botometer and at least one of our algorithms identified a user as a bot, the user was classified as such; conversely, the same principle was applied to the classification of humans. To account for the potential error in bot identification, we performed all our analysis with a varying baseline CAP score ranging between 0.60 and 0.75 (See more details in SI Bot Identification).

### Topic modeling

We first identified the themes emerging in the protest-related discourse with the increasingly popular bi-term topic models^50,77^ that learn topics by modeling word-word co-occurrence patterns^78^. After removing all retweets, to preprocess the data we used the nltk python package^79^ to remove stopwords, usernames, emojis and links from the tweets, and lemmtize and stemm every word.

We then trained bi-term topic models on our preprocessed data with the bitermplus package^80^. We set up a biterm topic model of all the tweets related to XR, which classified all tweets into 8 different topics. Based on the u_mass coherence scores^81^ (See Figure S5 in SI), we determined that 8 topics fits our dataset the best. After evaluating the meaning of topics, we dropped one topic whose keywords and content were too diverse to extract a meaningful media agenda from it. (See more details in SI Topic Modeling).

### Testing time-series relations

To test whether bots influence human activity or vica versa we employed Vector Error Correction Models (VECM). VECM can handle multivariate time series data that are likely cointegrated, which means that they share a stable long-term relationship despite short-term fluctuations^53^. Therefore it is more suitable compared to other methods, such as vector autoregressive (VAR) models, or granger causality tests it does not require stationary time data.

We created aggregated time series of the number of posts by bots and humans, then introduced a 30-minute time-lag between the number of tweets and the average sentiment by bots and by humans. The augmented Dickey-Fuller (ADF) test, the Kwiatkowski-Phillips-Schmidt-Shin (KPSS) tests and the Johansen test all suggested that all time series we tested were non-stationary (e.g., the mean or variant was not constant or there were seasonal fluctuations in the time series trend), but stationary for their first order difference. However, first-order differences of the time series were stationary, suggesting that they satisfied the prerequisite of VECM. The formula of VECM models is as follows:

$$\begin{aligned} \Delta Y_t = \alpha + \sum _{i=1}^{p-1} \Phi _i \Delta Y_{t-i} + \sum _{i=0}^{q} \Gamma _i \Delta Z_{t-i} + \beta _1 (Y_{t-1} - \beta X_{t-1}) + \epsilon _t \end{aligned}$$

where $\Delta Y_t$ represents the vector of first differences of *m* variables $Y_1, Y_2, \ldots , Y_m$ at time *t*. $\alpha$ stands for the constant term, $\Phi _i$ are coefficient matrices for the lagged first differences of *Y* variables up to $p-1$ lags, $\Gamma _i$ are coefficient matrices for the lagged first differences of *Z* control variables up to *q* lags, $\beta _i$ is a coefficient vector capturing the speed of adjustment (error correction term), $Y_{t-1}$ denotes the lagged level of the *Y* variables, $X_{t-1}$ denotes the lagged level of the *X* control variables, and $\epsilon _t$ represents the error term or residual at time *t*.

Apart from our main variables of interest (bot posts and human posts), we also included control variables in our VECM models. We control for the number of retweets of major news media communication relevant for our geographical coverage (US, UK, Canada): BBC News, The Guardian, The Telegraph, The Independent, Sky News, Channel 4 News, ITV News, Daily Mail, Financial Times, Metro Uk and CNN. It is primarily news media reports that prompt activists and social bots to react and engage, thus events of news media communications could be the third variable that influences the timing of both human and bot engagement.

If the number of posts by bots could predict (statistically significant coefficients in VECM models) that of humans, then we would infer that bots were directing humans’ attention, and vice versa. If no statistical significant was observed on both sides, or if the effect was mutual, following existing research practice, we concluded that bots and humans were driving the cascade together.^82–84^ (See SI Cascades, Table S1 for detailed information of the VECM test results on all identified cascades, and further statistical robustness tests.).

### Sentiment analysis

For sentiment analysis, the VADER package was used, which is an open source rule-based model and has been proven particularly effective for the classification of feelings in text from social media^85^. We used the raw text of tweets for sentiment analysis, as suggested by the package documentation. For each tweet, the algorithm assigns a sentiment score from $-1$ to $+1$, with $-1$ being the most negative, $+1$ the most positive, and 0 neutral. (See SI Figure S6 and S7 for the sentiment distribution of all tweets by exposed and matched users, and Section “Validating Sentiment Analysis”, SI table S21 for details on the accuracy of the VADER python package.)

### Matched sample

In order to understand how bots shape in the longer term (30 days after bot interaction) human tweeting activity and tweet sentiment related to XR, we created a sample of matched human users who did not interact with bots in our dataset to compare with ‘bot-exposed’ human users. We define bot interaction as very direct and active engagement of retweeting a bot (such as replying to a bot’s post), not merely seeing a post from a bot. We first identified a group of 506 users in our dataset as our exposed sample, the ones who directly interacted with bots by quoting or replying to bots. Then we calculated a similarity metric between all the ‘non-exposed’ human users and exposed users. Specifically, we calculated Eucledian distance based on the following metrics: statuses count, followers count, friends count, favorites count, listed count, followers growth (average number of followers increased on a daily basis), friends growth, favorites growth, listed growth, follower friend ratio. These traits were selected and/or calculated based on the user profile collected via Twitter API V1.2. The formula for the Euclidian distance is as follows, in which $p_n$ and $q_n$ means the $N_th$ trait for the sample and the matching:

$$\begin{aligned} d(p,q) = \root \of {(p_1 - q_1)^2 + (p_2 - q_2)^2 +... + (p_n - q_n)^2} \end{aligned}$$

It is worth mentioning that not all exposed users have matched users, and not all matched users were active during the time window in discussions related to XR. This is because the distribution of activity levels in online political communication is right skewed^34^; half of the users posted less than five times about XR in our data set. After dropping non-active potentially matched users, in total we had 184 matched users for 303 exposed users. If a user has more than one matched user, we include up to the top five matched users in our dataset.

Our grouping passed the parallel trend assumption test (Chi-squared, *p* = 0.076 for user sentiment, and *p* = 0.967 for amount of posts), indicating no significant difference in the slopes of the trends for the treated and control groups before the treatment. We also applied Propensity Score Matching (PSM) and Coarsened Exact Matching (CEM) which yield similar categories. (See SI Matching Design for details on matching-related robustness checks.)

### Identifying cascades

Cascades were identified by calculating the temporal density (the percentage of tweets that belong to a given topic in a given time period) of each topic. Based on previous studies on the life cycle of information cascades online^86,87^, the time window was specified as 1 hour.

To identify cascades, we identified bursty periods by calculating the Z-scores of the average topic density per hour for each topic, adopting methods by similar studies in social media^86,87^. We then filtered out all time units that had a Z-score larger than 2 (> 95% percentile) in any two or more consecutively two one hour time windows. Because the Z-score and the topic density could be high in time windows with only a few tweets, we also dropped those topics with no more than 50 tweets in at least one hourly time window. (See SI Cascades for detailed information on all identified cascades.)

### Support group categorization using ChatGPT

For our research objectives, we also categorize users’ opinions regarding the protests they engage in discussions about. To achieve this, we seek to determine whether users are in favor of or against the protests they discuss. This was measured using a scoring system ranging from −1 to 1, where −1 indicates complete opposition to the protest, 1 denotes strong support for the protest, and 0 signifies a neutral stance or unrelated discussion in their tweets. Subsequently, we employed a tri-category classification scheme for further analysis: scores between −1 and −0.1 are classified as “Anti-XR (Con protest)”, those from 0.1 to 1 as “Supporters (Pro protest),” and scores between −0.1 and 0.1 as “Neutral.”

The data used for this classification consists of users’ tweets from our dataset. We evaluated the opinion expressed in all interactions (human replies to bots) between our sample users and bots. Additionally, for each bot-exposed user and their matched counterparts, we classified their opinions based on all tweets from their timeline before bot interaction.

We employed OpenAI’s large language model (LLM), ChatGPT 3.5^88^, to classify users’ standpoints. This method has been raised and adopted in various studies^89,90^. For each user, we provided the model with an instruction prompt on how to classify their opinion toward climate change protests in general, along with the text to classify (users’ tweets), and the model outputed the aforementioned score. To generate the three opinion scores mentioned above, we used the full timeline for each user before bot interaction (for opinion before), the full timeline after bot interaction (for opinion after), and human replies to bots’ tweets during bot interaction (for opinion during interaction). (See SI Support Group Categorization, Table S4 for detailed information on prompt engineering and verification.)

### Difference-in-difference models

We applied difference-in-differences (DiD) analysis to assess the effects of the two-way treatment on human users who directly engaged with bots. DiD is a statistical model design that incorporates both a treatment and a control group. In this approach, we estimated the causal effect of treatment by analyzing time series data from both treatment and control groups. We compared the treatment effect of users who had direct interaction with bots and those who did not, 30 days after direct interaction.

The estimator and formula of a DiD model is as followed^54^:

$$\begin{aligned} Y_{it} = \alpha + \beta _1 \text {Treat}_{i} + \beta _2 \text {Post}_{t} + \beta _3 (\text {Treat}_{i} \times \text {Post}_{t}) + \epsilon _{it} \end{aligned}$$

In the estimator, $\text {Treat}_{i}$ is the key explanatory variable of differences in the treatment state, and $\text {Post}_{t}$ is the dummy temporal variable that says if it is before or after treatment.

For models estimating impacts on tweeting ‘amount’, we included days without any records of tweets (zero-tweet days) into our dataset for estimation. Because of the excessive number of zeros in the dependent variable in this case, we used zero-inflated negative binomial models to calculate the effect of bot interaction on the number of tweets. Since average daily sentiment is normally distributed, we used linear models to estimate bot impact.

We also controlled for variables that can provide alternative explanations for our findings. Our topic analysis revealed that bot activity is topic dependent and bots generate cascades, consequently influencing human communication. Throughout this process, bots might interact with humans multiple times. Therefore, we controlled for burstiness, the topic of the interaction, and the total number of bot interactions for each user. The sentiment of the interaction and the popularity of the original tweets could impact the level of activity in a thread. Longer threads may attract more bots, and we took these factors into account as well. An analysis of the demographic features (See SI Matching Design) of the sample and the matched groups indicated no statistically significant difference in gender. However, there was a significant difference in geographical locations. Consequently, we included location categories (UK and Ireland, Europe, USA, Australia and New Zealand, other locations) as an additional control variable.

In our models that compare the impact of bot interaction by support categories, we also controlled the support level of the interacted bot and their astroturf score. (See SI Tables S22–39 for full model tables).

## Supplementary Information

Supplementary Information.

## Data Availability

Analysis code and anonymized data created for the study will be available in a persistent GitHub repository upon publication. The link to the repository is as follows: https://github.com/lindali97/bot-cascade-climate-change. Please contact L.L. (corresponding author) in case anyone wants to request the data and code from this study.
