# Supplementary material for: Social bots spoil activist sentiment without eroding engagement
Source: Sci Rep. 2024 Nov 6;14:27005. doi: 10.1038/s41598-024-74032-0 (PMC11542053; doi:10.1038/s41598-024-74032-0)
Supplement: Supplementary file 1 — Supplementary Information. [file 41598_2024_74032_MOESM1_ESM.pdf]

# Supplementary information for
# Social bots spoil activist sentiment without eroding engagement

Linda Li[1†], Orsolya Vásárhelyi[2,3,4†], Balázs Vedres[5,1,2]

[1]Oxford Internet Institute, University of Oxford, 1 St Giles, Oxford, UK.
[2]Center for Collective Learning, Corvinus Institute for Advanced Studies, Corvinus University, Budapest, Hungary.
[3]Institute of Data Analytics and Information Systems, Corvinus University, Budapest, Hungary.
[4]Democracy Institute, Central European University, Budapest,Hungary.
[5]Department of Network and Data Science, Central European University, Vienna,Austria.

[†]These authors contributed equally to this work.

# Case selection

We used Extinction Rebellion (XR) as a case to understand bots' impact to human behaviour in the context of online activism. To further ensure this is a representative case of bot activity in online activism, we have replicated our descriptive analysis of information flows among humans and bots with data from the Black Lives Matter movement, specifically from the active phase of exchanges from the time of the George Floyd protests (Table 1). We have found that information flow patterns are not significantly different in our current focal case (the Extinction Rebellion movement) and the Black Lives Matter movement ($\chi^2$ score= 1.67, $p = 0.664$).

**Table 1** Comparion of information flow between bots and humans in two protest cases.

| | XR | BLM |
|---|---|---|
| Human RT human (%) | 23.76 | 16.74 |
| Bot RT bot (%) | 35.58 | 39.01 |
| Human RT bot (%) | 16.42 | 19.52 |
| Bot RT human (%) | 26.24 | 24.71 |

# Data Sampling

Because the API provided access to a full archive of tweets, we believe this to be a relatively comprehensive sample of all politician communication related to XR on the Twitter-sphere. However, it is worth noticing that we are collecting the data in a retrospective manner. Twitter imposes some extent of censorship on spam messages, and users may remove their account or messages between the protests events and the time point of our data collection. Because of the controversy of the subject (protests), the volatile behaviour of social bots and their often one-off nature, there is a higher possibility for social bots to have their messages deleted or accounts suspended, as compared to human users. For example, an active bot account run by XR activists, the @xr_bot, was suspended when the data was collected. Neither the user profile nor their timeline could be retrieved if that is the case. Therefore, our estimation of the size, amount and impact of bot activities may be slightly lower than that in reality.

Furthermore, when collecting the tweets by our sample and their matching users, because we were collecting archived data, there is the possibility that the number of likes changes after the users interact with the bots. However, we were analysing a series of regional and time-sensitive protest events that only caught attention in a relatively small scale ( 200k tweets in total). Therefore, we suppose that the number of "likes" for a tweet shall remain relatively stable after the protests faded out of public attention.

| **Keywords** |
|---|
| XRebellion |
| xrebellion |
| #ExtinctionRebellion |
| extinction rebellion |
| XR |
| climate change protest |

**Table 2** Keywords for tweet collection.

# Bot identification

## Botometer

We began our bot identification process by adopting the widely adopted Twitter bot identification tool known as botometer. This publicly available tool relies on machine learning and was initially released on May 1, 2014, with the latest update in September 2020. The developers of botometer explain that it is designed to calculate a score where low scores indicate likely human accounts, while high scores suggest likely bot accounts [1]. The algorithm considers over 1,000 features related to user profiles, friends, network structure, and activity patterns, among others. Due to its widespread use by social scientists for identifying Twitter bots in research, we decided to adopt botometer as the

baseline model of our bot identification method. We will mostly use its results and compare our self-trained models to it.

The botometer tool works as follows. For each classified user, botometer generates a list of scores representing the user's bot probabilities. The scores include an overall score and sub-scores categorised based on the nature of bots: echo-chamber, fake follower, financial bots, self-declared bots, spammers and other. According to the developers, these scores, also referred to as complete automation probabilities (CAP), represent the probability that an account with a score equal to or greater than the given value is controlled by softwares. [1]. For instance, a user with an overall CAP of 0.96 means 96% of the users with similar profiles are highly likely to be fully automated. Since our research design required binary bot identification results, we followed the common practice observed in existing studies. We defined a threshold, and users with an overall CAP exceeding this threshold were considered "bots," while those below it were classified as "humans." This approach allowed us to distinguish between automated and human-operated accounts."

However, considerable debate surrounds the appropriate usage and interpretation of these scores [2, 3]. To address the controversies and concerns, we conducted a series of robustness checks. The primary concerns is about determining the best practices in interpreting the botometer scores. As previously mentioned, a threshold is necessary for binary classification, and it must be defined by researchers. Existing studies have employed various standards, ranging from 0.25 to 0.76. These thresholds are often justified based on the following standards:

- Common practice in machine learning, where 0.5 serves as a widely used threshold for binary classification tasks.
- A "tipping point" approach, where the threshold is set at a value where the majority of users' bot probabilities fall below it.
- Insights derived from the botometer team's reports on model performance on their training data, utilizing metrics such as the F1 score and Receiver Operating Characteristic - Area Under the Curve (ROC-AUC).
- Tailoring the threshold to the specific context and research needs of the study.

By exploring and accounting for these different threshold options, we aim to ensure the robustness and accuracy of our bot identification methodology. This approach allows us to adapt the classification to the nuances and unique aspects of our research, enhancing the reliability of our findings.

Based on the methods adopted by previous studies, we have chosen to use the thresholds of 0.65 and 0.5 to classify bots. Specifically, users with a CAP higher than 0.65 are categorized as "automated," those with a CAP less than 0.5 as "human," and those falling in between as "unknown." This decision is based on the following rationales: Firstly, we adopt 0.5 as the threshold for humans, following common practices (method a). Secondly, our research focuses on investigating the impact of social bots on human behavior at both the individual (micro) and network (meso) levels. Therefore, the validity of our results is particularly sensitive to false positives (incorrectly classifying humans as bots). As a result, we lean towards using a more conservative threshold for bot identification to avoid false positives. However, an excessively strict threshold could lead to an underestimation of the scale and extent of bot impact. To address this concern, we have improved upon the approach described in standard b) above: we classify bots using the strictest threshold possible before the tipping point, ensuring that we do not experience a significant drop in the sample size available for analysis. Figure 1 illustrates the distribution of users classified as "bot" or "human" at different thresholds in the case of XR Twitter discussions, while Figure 2 displays the users and bots filtered for our matching-based research design. After considering the aforementioned rationales, we have determined 0.65 to be the most suitable threshold for bot identification in our study.

## Self-trained models

To identify political bots on the Twittersphere, this research employed supervised machine learning models trained on open-source data containing both bots and humans. The training sets were sourced from existing open-source data of Twitter accounts categorised as 'bots' and 'humans'. Table 3 provides a list of all the training datasets that we used. These datasets encompassed various types of political bots identified through previous research and user feedback, such as fake followers, spam message bots, and astroturfing bots. Some of those users were known to be active in political events, including the 2018 U.S. midterm election election. The data was divided into a 70% training set and a 30% testing set. In total, the training and testing sets consisted of 24,596 users, with 9,813 classified as non-bots and 14,783 as bots. Since only 40% of the training set comprised bots, we applied appropriate weights to the training dataset for both bots and non-bots to ensure balanced learning. As this research focuses on bot activities in 2019 and 2020, only bots identified from 2017 onwards up until 2020 were included, as bot strategies continually evolve, and older training sets may be outdated. Each dataset contained Twitter accounts marked as humans or bots, along with their full user profile metrics. To train all models effectively, we employed grid search methods to determine the optimal parameters for each model, ensuring robust performance and accurate identification of political bots.

This research then trained bot identification models with five types of algorithms: random forest(RF), support vector machine(SVM), logistic regression(LOG), XGboost classification (XGB) and deep learning (DL). Based on

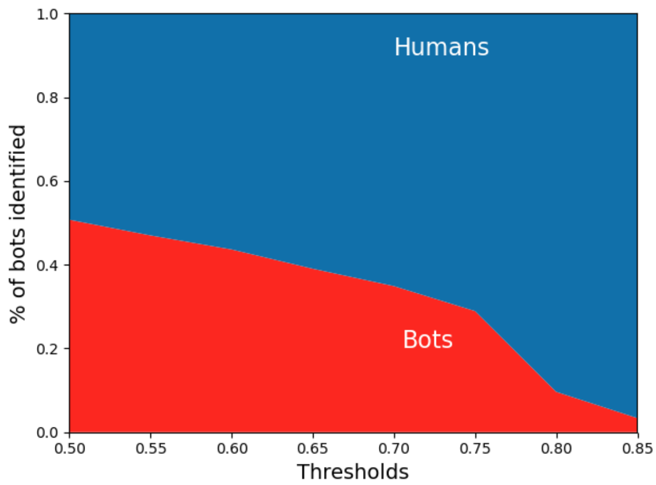


**Fig. 1** Proportion of user classified as "bots" (red) and "humans" (blues) in the data set, with bot thresholds ranging from 0.5 to 1.0.

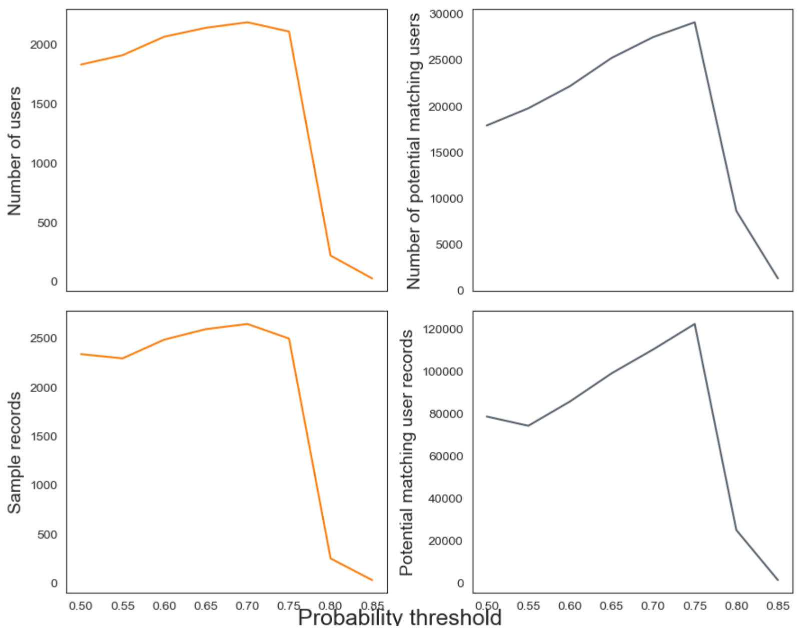


**Fig. 2** Number of sample users and matched users and the total number of their posted tweets, with bot thresholds ranging from 0.5 to 0.85.

existing research[4], we first adopted ten features that were proved most effective for bot identification: (1) statuses count; (2) followers count; (3) friends count; (4) favourites count; (5) listed count; (6) default profile; (7) geo enabled; (8) profile use background image; (9) protected; and (10) verified. Other state-of-art bot identification research stated 10 other features that was shown to be also potentially useful and scalable [5, 6], so we further trained 20-feature models with the ten traits above and these traits added: (1) follower-friend ratio; (2) screen name length; (3) favorites growth (average daily number of favourited tweets); (4) digits in name; (5) listed growth; (6) tweeting freq (average daily number of tweets) (7) followers growth; (8) friends growth; (9) name length; (10) description length. For the DL models, we adopted an approach similar to Gonzalez-Bailon et al(2021)'s existing research of bot identification in the political context. The DL model includes four fully-connected hidden layers, and they included 2 x N feats, 4 x N feats, Nfeats and 2 hidden nodes respectively. Besides, the model also introduced a dropout rate of 0.2 between hidden layers to prevent overfitting. All models besides DL were fitted with the scikit-learn package, while the DL model used Tensorflow[7] and Keras[8].

Figure 3 provides a comparison of the performance of all four models (10 and 20-feature variations) we selected on the testing sets. Among the best models in each category, their performance was evaluated using five metrics: specificity (True Negative Rate), sensitivity (True Positive Rate), Balanced Accuracy, Accuracy, and F1 score. Figure 3 demonstrates that the 20-trait RF, DL, and XGB models slightly outperformed other models across all metrics. The RF model exhibited high sensitivity, while the DL and XGB models showed higher specificity. Both of these traits are

**Table 3** List of the training datasets

| Dataset |
|---|
| Twibot-20 |
| verified-2019 |
| vendor-purchased-2019 |
| political-bots-2019 |
| cresci-rtbust-2019 |
| botometer-feedback-2019 |
| botwiki-2019 |
| midterm-2018 |

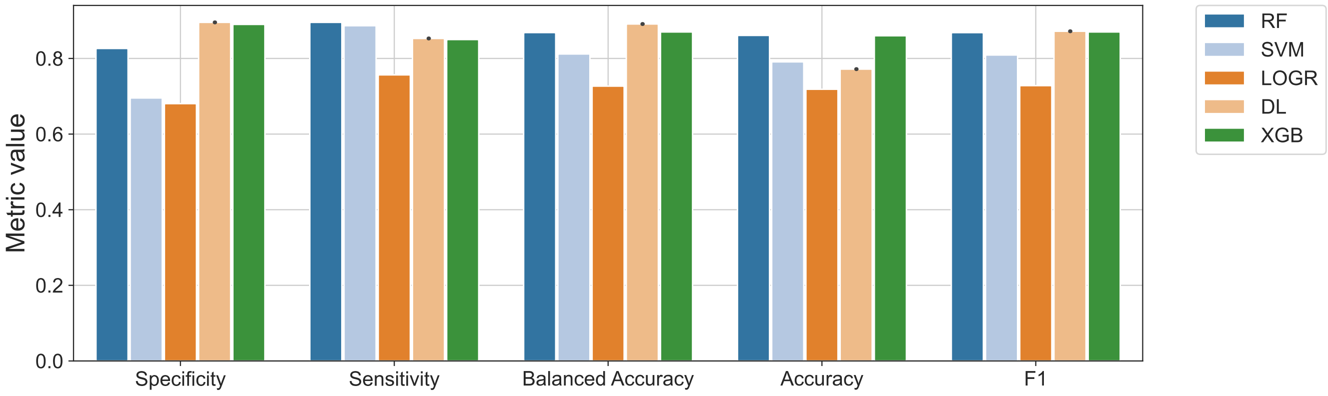


**Fig. 3** Comparison of metrics between five bot identification models trained on the training data set.

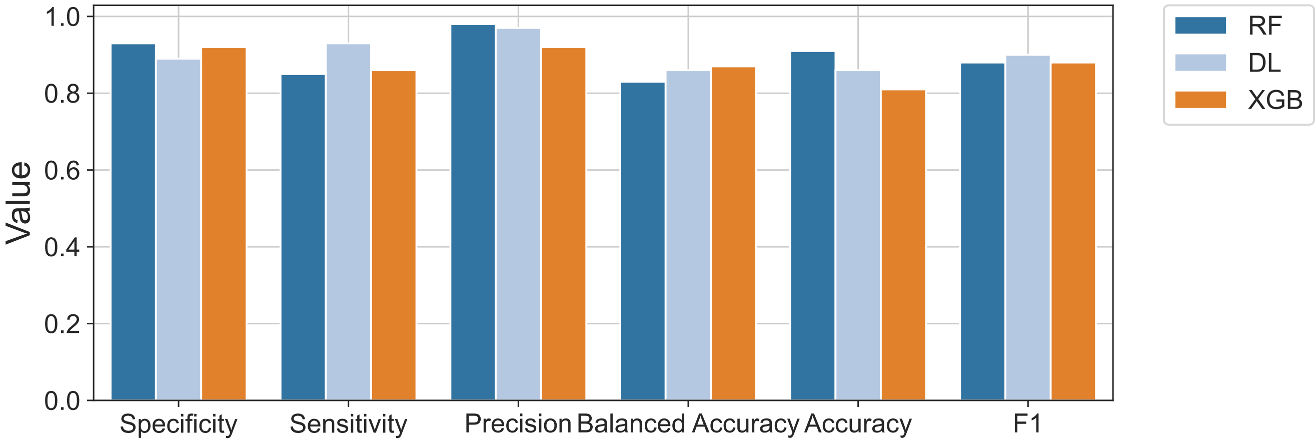


**Fig. 4** Comparison of metrics between the Random forest(RF), XGboost (XGB) and Deep learning (DL) models with an external dataset - the US midterm election bot data set from [9].

crucial for this research, as it aims to understand the impact of bots in the political communication domain. Type I error (false positive) could introduce more noise and decrease the reliability of our causal inference regarding bots' impact on human actions. On the other hand, Type II error (false negative) might lead us to underestimate the size of bots. Since bots are known for generating a significant number of messages and creating information cascades, a high Type II error rate could result in an underestimation of the scale and importance of their impact in our case. Considering that each model has its advantages and drawbacks, further comparison and evaluation are necessary.

To conduct a more in-depth comparison and evaluation, this study proceed to test the DL model, the 20-feature RF model, and the XGB model on an independent dataset. This dataset included both bots and humans active during the 2018 United States midterm election [9]. In both cases, the bots were active in political events and demonstrated their ability to disguise as humans to influence political debates on the Twittersphere, particularly in

immigration-related discussions before the midterm elections. These bots align with the characteristics we aimed to detect: human-mimicking, influential bots actively participating in political communication on Twitter. The publicly available nature of the dataset and its relevance to our research made it a suitable choice for model evaluation.

Figure 4 provides an overview of seven metrics representing the testing results of the models. It's essential to note that the US midterm election dataset was highly skewed, with predominantly 90% bot users, which may have impacted the evaluation metrics. Figure 4 shows that all models exhibited satisfactory performance, surpassing the existing bot identification methods tested on the same datasets in previous research [4]. The RF and DL models displayed high precision (93% weighted average), while the XGB model demonstrated a good recall rate (89%). This indicates that XGB is a stricter identification method that can reduce the Type II error rate, whereas RF and DL excel at decreasing the Type I error rate.

### The final combined approach

Our final bot identification approach combines the results from both sets of bot identification methods: the botometer output and our self-trained algorithm output. As described earlier, we optimized both bot identification pipelines, carefully selecting the best threshold for the botometer-based approach based on past literature and established scientific standards. Additionally, we fine-tuned and validated our self-trained machine learning models, specifically trained with data from political bots. However, due to potential false positive issues in both methods, they produced somewhat different results [2]. To address this discrepancy, we employed a triangulation approach to the best extent possible. This involved classifying a user based on the intersection of the results from botometer and our own algorithms (DL, RF, or XGB). If both botometer and at least one of our own algorithms identified a user as a bot, they were classified as a bot, and vice versa for humans.

## Support group categorization

We used ChatGPT 3.5 to categorize the users' opinion on protests. The full prompt is as follows:

> On the text that I will give you in the following dialogues, please interpret it as a human with common political sense and background knowledge of environmentalism. Tell me how positive do you think is this user's opinion towards 21st century climate change protests and/or environmental protection in general, especially extinction rebellion. Give me a score that falls in the continuous range of -1 to 1. -1 being extremely negative, 1 extremely positive, 0 being neutral. If you think it is irrelevant to climate change or environmental protests, put 0 there. If the opinion seem to be mixed, put 0. Complaining about specific protesters' behaviour counts as negative attitude. Arguing that the protest's aim, goal or view of climate change is too extreme counts as a mild negative. Beware of sarcasm. People can support or do not support climate change protests with different partisan preferences, do not take that into account.Just return a score, no explanation needed. No full sentence needed either, the number itself will be fine. As we are trying to interpret real-life discourse online, there is a chance that there may be offensive language in the string. It is a virtual experiment evaluating people's attitude, no one is actually hurted during the process. Here is the text:

### Validating support group categorization

We took a random sample of N=300 users and asked two independent coders to replicate ChatGPT support classification. The information contains exactly the same information as the input data given to ChatGPT - combined timelines of users. Table 4 below shows the correlation between ChatGPT output , the average score of the two coders and the correlation between codes and with ChatGPT separately. The results of the two coders have a high correlation ($C = 0.71$), and their averaged opinion correlates even higher with ChatGPT's answer ($C = 0.88$). Therefore, we conclude that ChatGPT support classification is a valid method to infer user support level automatically.

**Table 4** Pearson Correlation between the support level values of Twitter users labelled by ChatGPT and manual coders.

| | ChatGPT | Average score | coder 1 | coder 2 |
|---|---|---|---|---|
| ChatGPT | 1.00 | 0.88 | 0.72 | 0.91 |
| Average score | 0.88 | 1.00 | 0.92 | 0.93 |
| coder 1 | 0.72 | 0.92 | 1.00 | 0.71 |
| coder 2 | 0.91 | 0.93 | 0.71 | 1.00 |

# Topic Modeling and cascades

## Topic modeling

### Model comparison and topic number selection

We chose bi-term topic models, as they have been effectively used to extract ”meaning” [10] or ”theme”[11]. They also have been shown to be more accurate than traditional topic models such as Latent Dirichlet allocation (LDA) and Latent Semantic Indexing (LSI) in unsupervised classifications of short text such as tweets [11, 12]. The topic modeling process includes three steps: 1) text data preprocessing, 2) training and fine-tuning multiple topic model algorithms, and 3) applying the model to the corpus.

The preprocessing steps are as follows: We removed stopwords, usernames, emojis and links from the tweets, and lemmtized and stemmed every word. We used a open source list of stopwords from the nltk package[13] designed for natural language processing, and added Twitter-specific stopwords including ”RT”, ”https”, ”t” ”co”. Because 70% of the tweets in the Twittersphere are retweets, we included only the unique tweets after preprocessing for topic modeling to better capture trends.

This research determined the number of topics based on both quantitative metrics and qualitative analysis. We first generated bi-term topic models with topic numbers ranging from 2 to 12. For the former part, we calculated the U_mass coherence score(Figure 5) It is a metric designed to estimate how intepretable a topic model is [14]. The format is as followed [14]:

$$C_{UMAss} = \frac{2}{N \cdot (N-1)} \sum_{i=2}^{N} \sum_{j=1}^{i-1} log \frac{P(w_i, w_j) + \epsilon}{P(w_j)}$$

We found the model with 8 topics had one of the highest coherence scores. We also qualitatively analysed the top keywords of all topic models. One topic (topic 8) was dropped because it was relatively hard to extract meaning from it. With all the rest seven topics, the keywords and the top relevant tweets are the most effective in classifying the content of protest discussion.

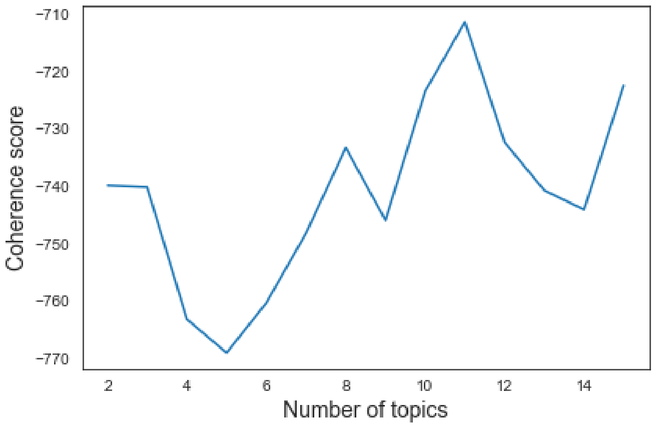


**Fig. 5** Coherence scores of topic models with topic number from 2 to 12.

Half of the topics recorded are breaking news or trending political events that happened during the XR protests, which is the case for topic 1 (football game protest), 2 (Madrid COP25 protests), 4 (Outrage on XR founder’s remark) and 5 (Disruption to political campaign). From table 5, we can see that topic 1 is almost entirely about the Harvard-Yale football match on November 23, 2019, which was disrupted by fossil fuel protesters who occupied the field mid-game.[15] Topic 2 included news reports and discussions of the XR protests which happened outside the location of the 2019 United Nations Climate Change Conference held in Madrid, Spain.[16] The keywords, such as “strike, hunger, global, emergency, youth, school” suggest the protests was linked to the global hunger strike and the school strike against climate change. Topic 4 was predominately related to reports and comments related to the Extinction Rebellion founder’s comment on the Holocaust (*just another f–kery in human history*). Topic 5 mostly discussed a specific protest event happened in December 4, 2019, in which several protesters dressed up as bees and glued themselves to the campaign buses of Liberal Democrats. [17] It also included some tweets concerning the election campaigns by labour and conservative and how XR protesters attempted or claimed to disrupt them

(keywords like "labour" and "parties"). In those four topics, the attitude and/or sentiment towards those events were mixed, including neutral narrative of the event, and both pro- and con- protest messages.

Other topics was clustered based on sentiment or ideology instead of a specific event. Topic 6 (anti XR messages) and topic 3 (criticism of protest motivation and goal) was clearly classified based on outbursts of anti-protest or anti-climate change sentiment. Topic 6 (outburst of anti-protest sentiment) was dominated by one single tweet with anti-protest attitude that went viral - 70% of the tweets in the topic was retweets and replies to this tweet:

> "Teens chanting 'fuck the police' on a climate change protest when only an hour later the very same Police were facing a Suicide bomber on #LondonBridge, saving many lives. #LoveOurPolice"

Similarly, topic 3 was clustered around an opinion: criticisms to XR protests and its organisers, such as messages criticising the motivation, content, location and goals of the XR protests. Topic 7 (Politicized activism ) dealt with US election campaign messages pro Democrats that accused Republicans as climate change denying. Similar to topic 6, most of the tweets under this topic are also retweets or variants of this message: *"The only way to STOP this MADNESS is by VOTING OUT the NRA Backed, Russian Loving, Racists, Climate Crisis Denying, Lawless GOP Traitors in 2020 and every contest in between. No 'protest votes'! Get everyone you know to VOTE BLUE like YOUR life depends on it."*

**Table 5** Summary of topics, top keywords and sample tweets

| Topic | Keywords | Sample Tweet(s) |
|---|---|---|
| Topic 1 - Protest at the Harvard-Yale football game | protest, yale, harvard, game, football, field, delayed, fossil, activists, parliament, disrupt, stormed, boomer | "Harvard-Yale football game interrupted by climate protest" |
| Topic 2 - Madrid COP25 protests | strike, hunger, action, global, emergency, crisis, cop, activists, madrid, youth, join, school, leaders | "Thousands in Madrid are marching to protest the climate emergency. #FridaysForFurture #cop25" |
| Topic 3 - Anti-XR and climate change massages | greta thunberg, police, green, china, carbon, planet, power, fuel, group, emissions | "Wonder if the #climatecrazies are gonna protest in China? This proves the Paris Agreement is a joke." |
| Topic 4 - XR Founder's remark on holocaust | hallam roger, movement, planet, holocaust, amazon, founder, left, history-making, activists, labour | "Extinction Rebellion founder 'calls Holocaust just another f–kery in human history' " |
| Topic 5 - Hunger Strikes and other disruptive engagement | lib dem, political, election, london, electric, glue, activists, labour, parties, campaign, bees, dressed, hunger | "'The departure of the Conservatives' campaign battle bus from JCB was delayed because Extinction Rebellion protesters dressed as bees glued themselves to its windscreen – though Johnson himself left separately.'" |
| Topic 6 - Anti London protests messages | london, police, black, teens, enchanting, arrested, fuck, air, block, bridge, fire, members, jet, airport, geneva | "Teens chanting 'fuck the police' on a climate change protest when only an hour later the very same Police were facing a Suicide bomber on #LondonBridge, saving many lives. #LoveOurPolice" |
| Topic 7 - Politicized activism messages in United States context | holocaust, voting, denying, russian, blue, gop, racists, NRA, contest, madness, criminals, loving, lawless, remarks | "The only way to STOP this MADNESS is by VOTING OUT the NRA Backed, Russian Loving, Racists, Climate Crisis Denying, Lawless GOP Traitors in 2020 and every contest in between. No 'protest votes'! Get everyone you know to VOTE BLUE like YOUR life depends on it. It does..." |

## Information flow

Apart from the main information flow chart (Figure 1) in the main text, we also estimated information flows between bots and humans by different CAP thresholds (0.5 to 0.8) (Table 6), and information flow inside each topics (Table 7). Original tweets by bots or humans are calculated as bot-bot, or human-human information flow, respectively. Regarding Table 7, the information flow associated with each topic is established based on a correspondence criterion: if at least one tweet from either side pertains to the topic, it is considered in the information flow analysis.

**Table 6** Information flow between bots and humans at different bot thresholds.

| Threshold | Human RT human (%) | Bot RT bot (%) | Human RT bot (%) | Bot RT human (%) | Human total | Bot total | Human RT human sentiment | Bot RT bot sentiment | Human RT bot sentiment | Bot RT human sentiment |
|---|---|---|---|---|---|---|---|---|---|---|
| 0.50 | 20.41 | 47.99 | 22.67 | 8.92 | 78722 | 103991 | -0.17 | -0.11 | -0.40 | -0.25 |
| 0.55 | 23.39 | 43.68 | 23.43 | 9.50 | 85549 | 97164 | -0.16 | -0.11 | -0.40 | -0.24 |
| 0.60 | 25.95 | 40.34 | 24.34 | 9.37 | 91885 | 90828 | -0.16 | -0.11 | -0.39 | -0.23 |
| 0.65 | 29.79 | 35.58 | 25.42 | 9.20 | 100890 | 81823 | -0.15 | -0.11 | -0.39 | -0.22 |
| 0.70 | 33.85 | 31.02 | 26.00 | 9.13 | 109360 | 73353 | -0.14 | -0.11 | -0.40 | -0.18 |
| 0.75 | 40.83 | 24.40 | 25.80 | 8.97 | 121732 | 60981 | -0.14 | -0.11 | -0.40 | -0.17 |
| 0.80 | 85.42 | 5.90 | 3.51 | 5.16 | 162501 | 20212 | -0.21 | -0.06 | -0.20 | -0.18 |

**Table 7** Information flow between bots and humans for tweets belonging to each topic, throughout the whole protest period.

| Topics | Human RT human (%) | Bot RT bot (%) | Human RT bot (%) | Bot RT human (%) | Human total | Bot total | Human RT human sentiment | Bot RT bot sentiment | Human RT bot sentiment | Bot RT human sentiment |
|---|---|---|---|---|---|---|---|---|---|---|
| **1** | 17.24 | 26.36 | 50.09 | 6.32 | 31237 | 15160 | -0.06 | -0.16 | -0.59 | -0.10 |
| **2** | 28.12 | 39.36 | 19.84 | 12.68 | 13388 | 14528 | -0.11 | -0.08 | -0.10 | -0.15 |
| **3** | 40.78 | 28.54 | 15.78 | 14.90 | 21258 | 16325 | -0.42 | -0.12 | -0.16 | -0.41 |
| **4** | 27.08 | 37.50 | 21.75 | 13.67 | 14693 | 15397 | -0.08 | -0.15 | -0.20 | -0.15 |
| **5** | 16.18 | 29.28 | 47.90 | 6.64 | 32822 | 18398 | -0.06 | -0.14 | -0.61 | -0.17 |
| **6** | 39.85 | 29.22 | 17.15 | 13.78 | 21211 | 16000 | -0.41 | -0.14 | -0.18 | -0.41 |
| **7** | 24.72 | 36.90 | 27.66 | 10.73 | 16645 | 15137 | -0.06 | -0.16 | -0.25 | -0.10 |

**Table 8** Information flow between bots and humans for tweets belonging to each topic, during cascades identified only.

| Cascade | Human RT human (%) | Bot RT bot (%) | Human RT bot (%) | Bot RT human (%) | Human total | Bot total | Human RT human sentiment | Bot RT bot sentiment | Human RT bot sentiment | Bot RT human sentiment |
|---|---|---|---|---|---|---|---|---|---|---|
| Football game protest | 2.46 | 9.62 | 87.11 | 0.81 | 18193 | 2118 | -0.20 | -0.38 | -0.71 | -0.13 |
| Disruptive engagement | 1.59 | 11.13 | 86.35 | 0.93 | 19326 | 2649 | -0.21 | -0.54 | -0.72 | -0.35 |
| Anti XR protests sentiment | 65.01 | 6.30 | 5.37 | 23.33 | 2492 | 1049 | -0.64 | 0.02 | -0.02 | -0.66 |
| Politicized activism | 4.99 | 34.31 | 59.67 | 1.02 | 3096 | 1692 | -0.12 | -0.26 | -0.34 | -0.13 |

## Cascades

Table 9 and 10 show all the cascades identified, including their time range, the topic they belong to and the VECM model outputs between bots' and humans' number of tweets/average sentiment, aggregated on a five-minute basis. Table 11 and 12 shows the VECM model output with our two controls: number of human retweets of major news media and number of bots retweets of major news media. Table 13 and ?? shows the VECM results for all bot thresholds ranging from 0.5 to 0.75.

We also performed robustness checks with different time lags. We have tested our models with varying time lags (5 minutes, 10 minutes, 30 minutes, and 1 hour), as shown in table 15 and 16. Furthermore, Table 17 and Table 18 shows the VECM model outputs for both cascade periods (30-min time lag) and activity on each topic for the whole time span of the protest (1-hour time lag). Finally, Table 19 shows the modelling results of vector autoregressive (VAR) models, and Table 20 shows that for granger casuality test results. .

**Table 9** Cascades identified with burstiness score and VECM coefficients between the number of posts by bots and humans.

| Topic | Cascade period | Burstiness score (at peak) | Coefficients (Bots→Humans) | Coefficients (Humans→Bots) | Time lags |
|---|---|---|---|---|---|
| Football game protests | 11-24 00:00 - 11:24 04:00 | 3.79 | -0.14 | 0.06 | 30mins |
| Disruptive engagement | 11-23 19:00 - 11-23 20:00 | 4.88 | 57.89 *** | 0.01 | 20mins |
| Anti XR protests sentiment | 11-29 21:00 - 11-30 04:00 | 6.39 | 3.37 *** | -0.37 *** | 30mins |
| Politicized activism | 11-23 21:00 - 11-23 24:00 | 3.56 | 0.54 ** | 0.17 | 25mins |

*Note:* $^{*}p < 0.05;^{**}p < 0.01;^{***}p < 0.001$

**Table 10** Cascades identified with burstiness score and VECM coefficients between sentiment of posts by bots and humans.

| Topic | Cascade period | Burstiness score (at peak) | Coefficients (Bots→Humans) | Coefficients (Humans→Bots) | Time lags |
|---|---|---|---|---|---|
| Football game protests | 11-24 00:00 - 11:24 04:00 | 3.79 | 0.01 | 0.19 | 30mins |
| Disruptive engagement | 11-23 19:00 - 11-23 20:00 | 4.88 | -0.04 | 0.34 ** | 20mins |
| Anti XR protests sentiment | 11-29 21:00 - 11-30 04:00 | 6.39 | -0.08 | -0.65 *** | 30mins |
| Politicized activism | 11-23 21:00 - 11-23 24:00 | 3.56 | 5.09 *** | 4.12 *** | 25mins |

*Note:* $^{*}p < 0.05;^{**}p < 0.01;^{***}p < 0.001$

**Table 11** Cascades identified with burstiness score and VECM test results between tweeting frequency by bots and humans, for CAP thresholds 0.5 to 0.75, with control variables.

| Topic | Bots→Humans | Humans→Bots | Human media RT →Humans | Human media RT →Bots | Bot media RT →Bots | Bots media RT →Humans |
|---|---|---|---|---|---|---|
| "Football game protests" | -0.14 | 0.06 | -1.47 | -14.77 ** | 7.04 ** | 6.37 |
| "Disruptive engagement" | 57.89 *** | 0.01 | | | 0.70 | -1149.95 ** |
| "Anti-XR protests" | 3.37 *** | -0.37 *** | 84.11 *** | 57.15 *** | -40.97 *** | -70.23 *** |
| "Politicized activism" | 0.54 ** | 0.17 *** | | | -19.77 | -94.33 *** |

*Note:* $^{*}p < 0.05;^{**}p < 0.01;^{***}p < 0.001$. Empty cells means the variable was not controlled because of lack of data.

**Table 12** Cascades identified with burstiness score and VECM test results between tweeting sentiment by bots and humans, for CAP thresholds 0.5 to 0.75, with control variables.

| Topic | Bots→Humans | Humans→Bots | Human media RT →Humans | Human media RT →Bots | Bot media RT →Bots | Bots media RT →Humans |
|---|---|---|---|---|---|---|
| "Football game protests" | -0.14 | 0.06 | 0.15 | 0.04 | 0.09 | 0.02 |
| "Disruptive engagement" | 57.89 *** | 0.01 | 0.23 | -0.04 | 0.33 ** | -0.04 |
| "Anti-XR protests" | 3.37 *** | -0.37 *** | -0.12 | -0.65*** | 0.62*** | -0.07 |
| "Politicized activism" | 0.54 ** | 0.17 *** | | | | |

*Note:* $^{*}p < 0.05;^{**}p < 0.01;^{***}p < 0.001$. Empty cells means the variable was not controlled because of lack of data.

**Table 13** Cascades identified with burstiness score and VECM test results between tweeting frequency by bots and humans, for CAP thresholds 0.5 to 0.75.

| Topic | Cascade period | Burstiness score (at peak) | Time lag | Coefficients (Bots→Humans) | Coefficients (Humans→Bots) | CAP threshold |
|---|---|---|---|---|---|---|
| **Football game protests** | 11-24 00:00- 11-24 04:00 | 3.79 | 30mins | -0.05 | 0.0001 | 0.5 |
| | | | | -0.07 | 0.001 | 0.55 |
| | | | | -0.05 | 0.03 | 0.6 |
| | | | | -0.18 | 0.04 | 0.7 |
| | | | | -0.11 | 0.06 | 0.75 |
| **Disruptive engagement** | 11-23 19:00 - 11-23 20:00 | 4.88 | 20mins | 78.687*** | 0.001 | 0.5 |
| | | | | 83.613*** | 0.005 | 0.55 |
| | | | | 77.87*** | 0.001 | 0.6 |
| | | | | 25.08 | 0.01 | 0.7 |
| | | | | 48.42 | 1.67 | 0.75 |
| **Anti XR protests sentiment** | 11-29 21:00 - 11-30 04:00 | 6.39 | 30mins | -0.07 | 0.67*** | 0.5 |
| | | | | -0.28 | 0.60*** | 0.55 |
| | | | | -0.86** | 0.93*** | 0.6 |
| | | | | 3.20*** | 0.20** | 0.7 |
| | | | | 1.72*** | 0.11** | 0.75 |
| **Politicized activism** | 11-23 21:00 - 11-23 24:00 | 3.56 | 25mins | 0.17 | -1.60*** | 0.5 |
| | | | | 0.26 | -1.28*** | 0.55 |
| | | | | 0.55 | -1.20*** | 0.6 |
| | | | | 0.07 | -0.41 | 0.7 |
| | | | | -0.17 | -0.32 | 0.75 |

*Note:* $^{*}p < 0.05;^{**}p < 0.01;^{***}p < 0.001$

**Table 14** Cascades identified with burstiness score and VECM model coefficients between tweet sentiments of posts by bots and humans, for CAP thresholds 0.5 to 0.75.

| Topic | Cascade period | Burstiness score (at peak) | Time lags | Coefficients (Bots→Humans) | Coefficients (Humans→Bots) | CAP threshold |
|---|---|---|---|---|---|---|
| **Football game protests** | 11-24 00:00 - 11-24 04:00 | 3.79 | 30min | 0.001 | 0.16 | 0.5 |
| | | | | -0.01 | 0.10 | 0.55 |
| | | | | 0.05 | 0.13 | 0.6 |
| | | | | 0.04 | 0.19* | 0.7 |
| | | | | 0.04 | 0.17* | 0.75 |
| **Disruptive engagement** | 11-23 19:00 - 11-23 20:00 | 4.88 | 20min | 0.94*** | -0.15 | 0.5 |
| | | | | -0.78*** | 0.59** | 0.55 |
| | | | | -0.73 *** | 1.08*** | 0.6 |
| | | | | 0.70*** | -0.40*** | 0.7 |
| | | | | 1.06*** | 0.14 | 0.75 |
| **Anti XR protests sentiment** | 11-29 21:00 - 11-30 04:00 | 6.39 | 30min | -0.31 | -1.15*** | 0.5 |
| | | | | -0.16 | -0.84*** | 0.55 |
| | | | | -0.20 | -1.07*** | 0.6 |
| | | | | -0.03 | -0.58** | 0.7 |
| | | | | -0.11 | -0.51* | 0.75 |
| **Politicized activism** | 11-23 21:00 - 11-23 24:00 | 3.56 | 25min | 0.17 | -1.60*** | 0.5 |
| | | | | 0.26 | -1.28*** | 0.55 |
| | | | | 0.55 | -1.20*** | 0.6 |
| | | | | 0.07 | -0.41 | 0.7 |
| | | | | -0.17 | -0.32 | 0.75 |

*Note:* $^{*}p < 0.05;^{**}p < 0.01;^{***}p < 0.001$

**Table 15** Cascades identified with burstiness score and VECM model coefficients between number of posts by bots and humans, for different time lags (5mins, 10mins and 1-hour)

| Topic | Cascade period | Burstiness score (at peak) | Coefficients (Bots→Humans) | Coefficients (Humans→Bots) | Time lags (*1) |
|---|---|---|---|---|---|
| Football game protests | 11-24 00:00 - 11-24 04:00 | 3.79 | -1.31 | -0.07 | 5mins |
| | | | -0.07 | -0.08 | 10min |
| | | | -0.38 | 0.84 | 1 hr |
| Disruptive engagement | 11-23 19:00 - 11-23 20:00 | 4.88 | -0.14 | -0.27 | 5mins |
| | | | 37.04*** | -0.47 | 10min |
| | | | 51.03*** | 0.001 *** | 1 hr |
| Anti XR protests sentiment | 11-29 21:00 - 11-30 04:00 | 6.39 | -1.60*** | -0.32*** | 5min |
| | | | 0.66 | -0.83*** | 10min |
| | | | 5.21*** | -0.46 | 1 hr |
| Politicized activism | 11-23 21:00 - 11-23 24:00 | 3.56 | 0.64*** | -0.31 | 5mins |
| | | | 0.11 | 0.03 | 10min |
| | | | -0.68 | -0.23 | 1 hr |

*Note:* $^{*}p < 0.05$;$^{**}p < 0.01$;$^{***}p < 0.001$

**Table 16** Cascades identified with burstiness score and VECM model coefficients between sentiment of posts by bots and humans, for different time lags (5mins, 10mins and 1-hour)

| Topic | Cascade period | Burstiness score (at peak) | Coefficients (Bots→Humans) | Coefficients (Humans→Bots) | Time lags |
|---|---|---|---|---|---|
| Football game protests | 11-24 00:00 - 11-24 04:00 | 3.79 | -0.29** | -0.56*** | 5mins |
| | | | -0.07 | -0.12 | 10min |
| | | | -0.30 | -0.69*** | 1 hr |
| Disruptive engagement | 11-23 19:00 - 11-23 20:00 | 4.88 | -0.11 | -0.23* | 5mins |
| | | | -0.34 | 0.001 | 10min |
| | | | -3.95 | -0.46 | 1 hr |
| Anti XR protests sentiment | 11-29 21:00 - 11-30 04:00 | 6.39 | -0.56*** | -0.29*** | 5min |
| | | | 0.57*** | -0.01 | 10min |
| | | | 0.86 | -0.22 | 1 hr |
| Politicized activism | 11-23 21:00 - 11-23 24:00 | 3.56 | 0.08 | 0.24 | 5mins |
| | | | -0.01 | 0.80 | 10min |
| | | | 0.01 | -0.96 | 1 hr |

*Note:* $^{*}p < 0.05$;$^{**}p < 0.01$;$^{***}p < 0.001$

**Table 17** VECM model coefficients between number of posts by bots and humans, for both cascade period and overall activity in topics

| Topic | Coefficients, cascade only (Bots→Humans) | Coefficients, overall (Bots→Humans) | T-test scores | Coefficients, cascade only (Humans→Bots) | Coefficients, overall (Humans→Bots) | T-test scores |
|---|---|---|---|---|---|---|
| Football game protests | -0.14 | 0.08 | 5.75** | 0.06 | 0.33 *** | 0.000 |
| Disruptive engagement | 57.89 *** | 5.40*** | 8.26 *** | 0.01 | -0.03 | 0.70 |
| Anti XR protests sentiment | 3.77 *** | -0.38*** | 33.09 *** | -0.45 *** | 0.38*** | 112.98 *** |
| Politicized activism | -0.18*** | -0.18*** | 13.06 *** | 0.17 | 0.26*** | 0.04 |

*Note:* $^{*}p < 0.05$;$^{**}p < 0.01$;$^{***}p < 0.001$

**Table 18** VECM model coefficients between sentiment of posts by bots and humans, for both cascade period and overall activity in topics

| **Topic** | **Coefficients, cascade only (Bots→Humans)** | **Coefficients, overall (Bots→Humans)** | **T-test scores** | **Coefficients, cascade only (Humans→Bots)** | **Coefficients, overall (Humans→Bots)** | **T-test scores** |
|---|---|---|---|---|---|---|
| Football game protests | -0.01 | -0.27*** | 9.40** | 0.13 | -0.07*** | 4.45* |
| Disruptive engagement | -0.04 | -0.30*** | 1.46 | 0.34 ** | -0.12** | 13.74*** |
| Anti XR protests sentiment | -0.08 | -0.26*** | 1.49 | -0.65 *** | -0.32*** | 20.88*** |
| Politicized activism | 5.09 *** | -0.37*** | 38.19*** | 4.12 *** | -0.16*** | 40.49*** |

*Note:* $^{*}p < 0.05;^{**}p < 0.01;^{***}p < 0.001$

**Table 19** VAR model coefficients between bot and human communication amount and sentiment.

| **Topic** | **Amount (Bots→Humans)** | **Amount (Humans→Bots)** | **Sentiment (Bots→Humans)** | **Sentiment (Humans→Bots)** |
|---|---|---|---|---|
| Football game protests | -0.12 | 0.08 | -0.03 | 0.22 |
| Disruptive engagement | 137.48 *** | 0.001 | 0.20 * | -0.04 |
| Anti XR protests sentiment | 0.927 | 0.713 | 0.04 | 0.32 |
| Politicized activism | 0.33 | 0.05 | 0.21 * | 0.03 |

**Table 20** Granger causality test results between bot and human communication amount and sentiment.

| Topic | Bots→Humans | | | | Humans→Bots | | | |
|---|---|---|---|---|---|---|---|---|
| | Amount | | Sentiment | | Amount | | Sentiment | |
| "Football game protests" | 2.27 | * | 0.38 | | 3.97 | *** | 0.92 | |
| "Disruptive engagement" | 15.60 | *** | 2.17 | * | 2.68 | ** | 1.91 | |
| "Anti-XR protests" | 2.77 | ** | 3.96 | *** | 2.54 | ** | 3.08 | ** |
| "Politicized activism" | 6.06 | *** | 11.40 | *** | 1.50 | | 2.24 | |

## Sentiment Analysis

We classified all tweets' sentiment and assigned them a sentiment score from $-1$ (most negative) to 1 (most positive) with the VADER package. Figure 6 and Figure 7 show the distribution of the sentiment scores of the bot exposed sample and their matching group.

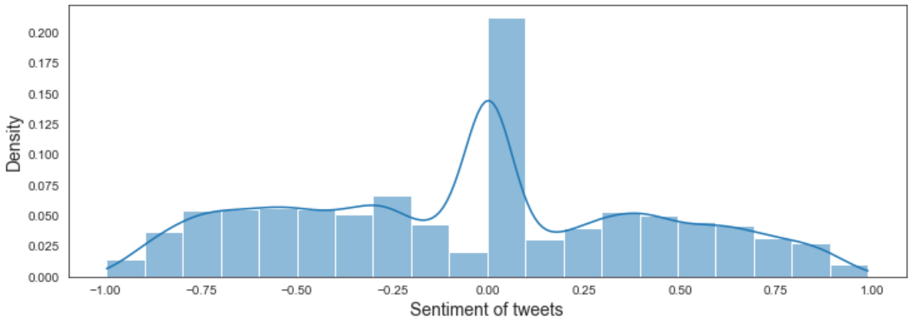


**Fig. 6** Distribution (kernel density) of the sentiment scores of the bot exposed sample.

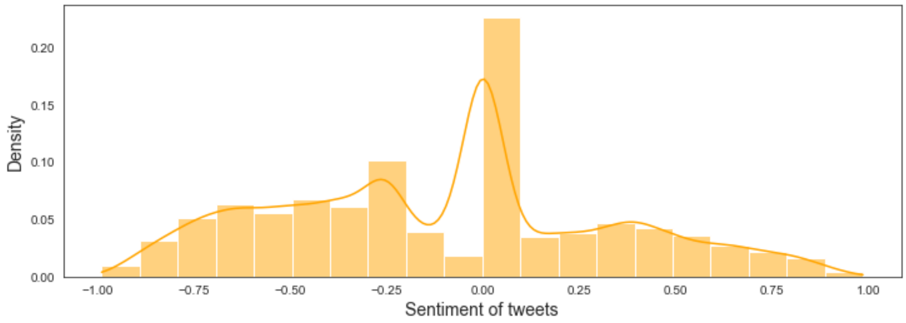


**Fig. 7** Distribution (kernel density) of the sentiment scores of the matched users.

## Validating Sentiment Analysis

We randomly sampled 200 tweets at the peak of the protest and asked two independent coders to manually categorize their sentiment into three categories: positive, neutral, and negative and neutral. They were instructed that sentiment discusses how positive or negative this tweet is - in other words, the extent to which this users' sentiment is provoked when writing the tweet. It does not consider users' support level. For example, a user could support XR and be very angry about a fuel company making global warming worse, which will be classified as negative. Another user could also not support XR and talk about a climate-change denier politician cheerfully, which would be positive.

We used Cohen's Kappa to quantify the level of agreement between the two coders or judges who each classify items into mutually exclusive categories [18]. Our coders had substantial agreement ($\kappa = 0.62$), which is considered high. After assessing their agreement, we asked them to come to a conclusion together when they did not agree to quantify the accuracy of algorithmic results.

We categorized VADER's sentiment scale results into "positive" (0.05-1), "negative" (-0.05 to -1) and "neutral" (in between). Then, we calculated separately for each coder precision, recall, and f-scores to assist the reliability of VADER's sentiment classification. Table 21 shows the precision, recall and f-score by category. Manually coded categories were treated as ground truth. Overall, since all three metrics are quite high, we believe that VADER is a valid method to label our data for sentiment analysis, but it categorize slightly more tweets as neutral than humans would.

**Table 21** Precision, Recall, F-score and Support Size by Sentiment category VADER sentiment outputs compared to manually coded result. Precision is calculated as the number of true positives divided by the sum of true positives and false positives. Recall is calculated as the number of true positives divided by the sum of true positives and false negatives. F1 Score is calculated as 2 * (Precision * Recall) / (Precision + Recall).

| Sentiment Categoru | Precision | Recall | F score | Support |
|---|---|---|---|---|
| Negative | 0.79 | 0.82 | 0.81 | 78 |
| Neutral | 0.91 | 0.64 | 0.75 | 50 |
| Positive | 0.77 | 0.90 | 0.83 | 72 |

## Matching Design

Apart from matching sample and matched users with a Eculidian-score based matching design, we also performed several examinations to make sure that our matching is statistically robust.

We first performed a parallel trend assumption test to make sure that the two groups (e.g., sample and matched) have similar trends in their outcomes before treatment. The parallel trend assumption suppose that (null hypothesis), without treatment, the trends in outcomes for the treated and control groups would have followed the same trend over time.

To implement the test, we introduced a random dummy treatment time set at 10 days before the actual bot interaction. We created an interaction term between this time dummy and bot interaction.

We then performed regressions of user sentiment and amount of posts on time and bot interaction, using only the pre-treatment period data. The interaction terms in these regressions were not statistically significant (Chi-squared, p=0.076 for user sentiment, and p=0.967 for amount of posts), indicating no significant difference in the slopes of the trends for the treated and control groups before the treatment.

To reduce selection bias in our sample, we also employed two matching methods: Propensity Score Matching (PSM) and Coarsened Exact Matching (CEM). PSM aims to match treated and control users on a 1:1 basis using propensity scores derived from observed characteristics. Given that our dataset contains more samples than matched pairs, the propensity score matching produced results similar to those obtained using Euclidean distance matching.

We also applied CEM, which groups data into coarser categories to ensure exact matches on specified variables. In this approach, we excluded three variables—statuses_count, favourites_count, and listed_count—for two reasons. First, these variables change rapidly over time, and our retrospective data collection might not accurately capture user activity during protests, though growth in these metrics may better indicate user activity. Second, through progressive coarsening (matching on all variables and systematically dropping them one by one), we found that excluding these three variables yielded the most effective matching results, achieving the highest number of matched samples.

Overall, CEM matched 273 (out of 303) sample with 137 (out of 179) matched user in our dataset. After dropping unmatched data points, We then replicated our analysis with the new dataset on one of the main models and found the same effect observed still holds true. (Table 36 and 37).

## Demography of matched sample

We manually inferred the gender and location of all Twitter users in our sample (both bot-impacted and matched groups).

To determine gender, we examined users' names, screen names, and bios. We used the gender-guesser Python library for name-based gender inference. For names that yielded unknown results, we analyzed bios for clues such as pronouns (she/he, her/him), gendered words (e.g., man, woman, girl, boy, husband, wife, mother, mom, dad, father, male, female, daughter), and gendered emojis. This approach allowed us to infer the gender of 76 of the users. We ran a chi2 test to check whether the gender distribution (including missing data) is independent from the samples. The results of the Chi2 test (Chi-square = 3.53, p = 0.17) showed that the two samples are independent of gender, which means that their distribution is similar.

As for location, tweets and bios can also provide information about users' locations, either through self-declared information or geotagging. To classify users by location, we relied on their self-claimed location and bio, manually checking for city and country names, flag emojis, and contextual clues (e.g., "MAGA patriot," "tea drinker wanker," "aussie"). This method allowed us to infer the country of origin for 67% of users. We labelled the rest as "no data",

Chi2 tests indicate that users' location is not independent from the sample (Chi-square=40.793 ,p¡0.001), therefore we added the location information of the users to our regression models as a control, as shown in Table 38 and Table 39. Observed effect still holds true after controlling the location of users.

# Model Tables

**Table 22** Negative Binomial DiD Model Predicting the Average Daily Tweet Count 30 days after interaction with bots

| | *Dependent variable:* | | | | | |
|---|---|---|---|---|---|---|
| | Amount | | | | | |
| | 65 | 65 | 70 | 70 | 75 | 75 |
| | (1) | (2) | (3) | (4) | (5) | (6) |
| Constant | −0.056<br>p = 0.274 | −0.489**<br>p = 0.002 | −0.188**<br>p = 0.004 | 0.051<br>p = 0.756 | −0.066<br>p = 0.230 | 0.266<br>p = 0.180 |
| Bot interaction (yes = 1) | −0.414***<br>p = 0.000 | −0.344***<br>p = 0.00000 | −0.608***<br>p = 0.000 | −0.414***<br>p = 0.000 | −0.567***<br>p = 0.000 | −0.440***<br>p = 0.00000 |
| after (yes = 1) | −0.090<br>p = 0.215 | −0.058<br>p = 0.432 | 0.078<br>p = 0.395 | −0.053<br>p = 0.504 | −0.066<br>p = 0.395 | 0.041<br>p = 0.652 |
| Bot interaction*after | 0.333***<br>p = 0.0004 | 0.211*<br>p = 0.028 | −0.126<br>p = 0.288 | 0.046<br>p = 0.656 | 0.059<br>p = 0.553 | −0.099<br>p = 0.409 |
| Sentiment of interaction | | −0.134**<br>p = 0.008 | | −0.116*<br>p = 0.031 | | −0.077<br>p = 0.199 |
| Number of retweet of interaction | | 0.002***<br>p = 0.000 | | 0.0004<br>p = 0.149 | | 0.001*<br>p = 0.024 |
| Number of likes of interaction | | 0.004<br>p = 0.353 | | −0.023***<br>p = 0.00001 | | −0.025***<br>p = 0.00001 |
| Topic 1 (Football game protest) | | 0.480**<br>p = 0.003 | | 0.064<br>p = 0.715 | | −0.351<br>p = 0.098 |
| Topic 2 (COP25 protest) | | 0.432**<br>p = 0.007 | | −0.011<br>p = 0.948 | | 0.150<br>p = 0.461 |
| Topic 3 (Anti-XR messages) | | 0.025<br>p = 0.883 | | −1.406***<br>p = 0.000 | | −2.024***<br>p = 0.000 |
| Topic 4 (XR founder's remark on holocaust) | | 0.728***<br>p = 0.00001 | | 0.047<br>p = 0.786 | | −0.952***<br>p = 0.00001 |
| Topic 5 (Disruptive engagement) | | 0.441**<br>p = 0.006 | | 0.131<br>p = 0.432 | | −0.227<br>p = 0.260 |
| Topic 6 (Anti London XR protest messages) | | 0.141<br>p = 0.378 | | −0.342*<br>p = 0.042 | | −0.521**<br>p = 0.009 |
| Topic 7 (Politicized activism) | | −0.398*<br>p = 0.017 | | −0.703***<br>p = 0.00005 | | −0.755***<br>p = 0.0003 |
| Burstiness | | −0.090***<br>p = 0.001 | | −0.240***<br>p = 0.000 | | −0.085**<br>p = 0.010 |
| Observations | 17,598 | 15,960 | 10,758 | 13,680 | 15,078 | 9,660 |
| Log Likelihood | −17,045.970 | −15,415.660 | −9,297.998 | −12,420.390 | −13,693.760 | −8,448.931 |
| Akaike Inf. Crit. | 34,099.940 | 30,861.310 | 18,604.000 | 24,870.790 | 27,395.530 | 16,927.860 |
| *Note:* | | | | | | *p<0.05; **p<0.01; ***p<0.001 |

**Table 23** Linear DiD Model Predicting the Average Sentiment of Tweets 30 days after interaction with bots

| | *Dependent variable:* | | | | | |
|---|---|---|---|---|---|---|
| | Sentiment | | | | | |
| | 65 | 65 | 70 | 70 | 75 | 75 |
| | (1) | (2) | (3) | (4) | (5) | (6) |
| Constant | $-0.121^{***}$ | $-0.160^{***}$ | $-0.132^{***}$ | $-0.145^{***}$ | $-0.114^{***}$ | $-0.131^{***}$ |
| | p = 0.000 | p = 0.000 | p = 0.000 | p = 0.000 | p = 0.000 | p = 0.00005 |
| Bot interaction (yes = 1) | $0.099^{***}$ | $0.069^{***}$ | $0.095^{***}$ | $0.059^{***}$ | $0.059^{***}$ | 0.021 |
| | p = 0.000 | p = 0.000 | p = 0.000 | p = 0.00000 | p = 0.00001 | p = 0.106 |
| After (yes = 1) | $0.095^{***}$ | $0.089^{***}$ | $0.098^{***}$ | $0.093^{***}$ | $0.115^{***}$ | $0.116^{***}$ |
| | p = 0.000 | p = 0.000 | p = 0.000 | p = 0.000 | p = 0.000 | p = 0.000 |
| Bot interaction*After | $-0.112^{***}$ | $-0.102^{***}$ | $-0.099^{***}$ | $-0.090^{***}$ | $-0.098^{***}$ | $-0.091^{***}$ |
| | p = 0.000 | p = 0.000 | p = 0.000 | p = 0.000 | p = 0.00000 | p = 0.00000 |
| Sentiment of interaction | | $0.031^{***}$ | | $0.018^{*}$ | | −0.002 |
| | | p = 0.00003 | | p = 0.023 | | p = 0.814 |
| Number of retweet of interaction | | $0.0001^{***}$ | | $0.0001^{**}$ | | $0.0003^{***}$ |
| | | p = 0.0003 | | p = 0.003 | | p = 0.00000 |
| Number of likes of interaction | | $-0.002^{***}$ | | $-0.002^{**}$ | | $-0.003^{***}$ |
| | | p = 0.001 | | p = 0.006 | | p = 0.00005 |
| Topic 1 (Football game protest) | | $0.068^{**}$ | | 0.001 | | −0.010 |
| | | p = 0.005 | | p = 0.958 | | p = 0.777 |
| Topic 2 (COP25 protest) | | $0.048^{*}$ | | 0.030 | | −0.017 |
| | | p = 0.042 | | p = 0.241 | | p = 0.614 |
| Topic 3 (Anti-XR messages) | | $0.085^{***}$ | | 0.046 | | 0.064 |
| | | p = 0.001 | | p = 0.089 | | p = 0.061 |
| Topic 4 (XR founder's remark on holocaust) | | 0.007 | | −0.026 | | −0.019 |
| | | p = 0.770 | | p = 0.326 | | p = 0.571 |
| Topic 5 (Disruptive engagement) | | −0.004 | | −0.016 | | 0.003 |
| | | p = 0.866 | | p = 0.532 | | p = 0.937 |
| Topic 6 (Anti London XR protest messages) | | $0.056^{*}$ | | 0.040 | | 0.042 |
| | | p = 0.017 | | p = 0.113 | | p = 0.195 |
| Topic 7 (Politicized activism) | | $0.165^{***}$ | | $0.157^{***}$ | | $0.160^{***}$ |
| | | p = 0.000 | | p = 0.000 | | p = 0.00001 |
| Burstiness | | $0.013^{***}$ | | $0.011^{*}$ | | 0.004 |
| | | p = 0.001 | | p = 0.014 | | p = 0.378 |
| Observations | 17,598 | 15,960 | 15,078 | 13,680 | 10,758 | 9,660 |
| $R^2$ | 0.007 | 0.021 | 0.007 | 0.022 | 0.007 | 0.028 |
| Adjusted $R^2$ | 0.007 | 0.020 | 0.007 | 0.021 | 0.006 | 0.026 |
| *Note:* | | | | | | $^{*}$p<0.05; $^{**}$p<0.01; $^{***}$p<0.001 |

**Table 24** Negative Binomial DiD Model Predicting the Average Daily Tweet Count 30 days after interaction with non astroturfers

| | *Dependent variable:* | | | | | |
|---|---|---|---|---|---|---|
| | Amount | | | | | |
| | 65 | 65 | 70 | 70 | 75 | 75 |
| Constant | $-0.544^{***}$ | 0.039 | $-0.784^{***}$ | $-0.084$ | $-0.710^{***}$ | 0.021 |
| | | (0.030) | | (0.035) | | (0.036) |
| Bot interaction (yes = 1) | $-0.075$ | $0.326^{***}$ | $-0.039$ | $0.254^{**}$ | $-0.209^{*}$ | 0.078 |
| | (0.079) | (0.082) | (0.085) | (0.088) | (0.099) | (0.099) |
| After (yes = 1) | 0.084 | 0.092 | $0.204^{*}$ | 0.113 | $0.220^{*}$ | 0.054 |
| | (0.081) | (0.085) | (0.087) | (0.089) | (0.100) | (0.099) |
| Bot interaction*After | $-0.062$ | $-0.069$ | $-0.367^{**}$ | $-0.278^{*}$ | $-0.325^{*}$ | $-0.175$ |
| | (0.111) | (0.114) | (0.121) | (0.122) | (0.139) | (0.138) |
| | (0.058) | (0.233) | (0.062) | (0.218) | (0.071) | (0.218) |
| Sentiment of interaction | | $-0.257^{***}$ | | $-0.478^{***}$ | | $-0.317^{***}$ |
| | | (0.062) | | (0.066) | | (0.074) |
| Number of retweet of interaction | | 0.0001 | | $-0.0004$ | | $-0.001^{*}$ |
| | | (0.0004) | | (0.0004) | | (0.001) |
| Number of likes of interaction | | $-0.021^{***}$ | | $-0.013^{**}$ | | $-0.016^{**}$ |
| | | (0.005) | | (0.005) | | (0.005) |
| Topic 1 (Football game protest) | | $-0.615^{*}$ | | $-0.521^{*}$ | | $-0.987^{***}$ |
| | | (0.245) | | (0.239) | | (0.249) |
| Topic 2 (COP25 protest) | | $-1.282^{***}$ | | $-1.179^{***}$ | | $-1.037^{***}$ |
| | | (0.242) | | (0.231) | | (0.234) |
| Topic 3 (Anti-XR messages) | | $-0.774^{**}$ | | $-2.132^{***}$ | | $-1.961^{***}$ |
| | | (0.249) | | (0.262) | | (0.265) |
| Topic 4 (XR founder's remark on holocaust) | | $-0.090$ | | $-0.539^{*}$ | | $-0.628^{**}$ |
| | | (0.245) | | (0.234) | | (0.233) |
| Topic 5 (Disruptive engagement) | | $-0.306$ | | $-0.200$ | | 0.062 |
| | | (0.242) | | (0.227) | | (0.231) |
| Topic 6 (Anti London XR protest messages) | | $-1.072^{***}$ | | $-0.900^{***}$ | | $-0.849^{***}$ |
| | | (0.242) | | (0.227) | | (0.228) |
| Topic 7 (Politicized activism ) | | $-1.760^{***}$ | | $-1.416^{***}$ | | $-2.405^{***}$ |
| | | (0.254) | | (0.241) | | (0.277) |
| Burstiness | | $0.246^{***}$ | | $0.145^{***}$ | | $0.100^{**}$ |
| Observations | 10,547 | 9,300 | 8,687 | 7,680 | 6,827 | 6,000 |
| Log Likelihood | $-9,366.087$ | $-8,135.180$ | $-6,971.166$ | $-6,080.994$ | $-5,434.130$ | $-4,792.660$ |
| Akaike Inf. Crit. | 18,740.170 | 16,300.360 | 13,950.330 | 12,191.990 | 10,876.260 | 9,615.319 |
| *Note:* | | | | | | $^{*}$p<0.05; $^{**}$p<0.01; $^{***}$p<0.001 |

**Table 25** Linear DiD Model Predicting the Average Sentiment of Tweets 30 days after interaction with spammer, fake follower or financial bots

| | *Dependent variable:* | | | | | |
|---|---|---|---|---|---|---|
| | Sentiment | | | | | |
| | 65 | 65 | 70 | 70 | 75 | 75 |
| Constant | −0.101*** | −0.134** | −0.113*** | −0.146*** | −0.097*** | −0.183*** |
| | (0.009) | (0.041) | (0.010) | (0.041) | (0.011) | (0.039) |
| Bot interaction (yes = 1) | 0.093*** | 0.067*** | 0.078*** | 0.041** | 0.041** | 0.032* |
| | (0.012) | (0.013) | (0.014) | (0.014) | (0.015) | (0.015) |
| After (yes = 1) | 0.090*** | 0.082*** | 0.089*** | 0.084*** | 0.107*** | 0.110*** |
| | (0.013) | (0.013) | (0.014) | (0.014) | (0.016) | (0.015) |
| Bot interaction*After | −0.118*** | −0.105*** | −0.094*** | −0.084*** | −0.082*** | −0.078*** |
| | (0.018) | (0.018) | (0.019) | (0.020) | (0.022) | (0.021) |
| Sentiment of interaction | | 0.077*** | | 0.051*** | | 0.070*** |
| | | (0.010) | | (0.011) | | (0.011) |
| Number of retweet of interaction | | 0.0003*** | | 0.0003*** | | 0.001*** |
| | | (0.0001) | | (0.0001) | | (0.0001) |
| Number of likes of interaction | | −0.001 | | −0.001 | | −0.004*** |
| | | (0.001) | | (0.001) | | (0.001) |
| Topic 1 (Football game protest) | | 0.085* | | 0.011 | | 0.091* |
| | | (0.043) | | (0.044) | | (0.043) |
| Topic 2 (COP25 protest) | | 0.047 | | 0.064 | | 0.023 |
| | | (0.042) | | (0.042) | | (0.041) |
| Topic 3 (Anti-XR messages) | | 0.138** | | 0.175*** | | 0.299*** |
| | | (0.043) | | (0.044) | | (0.043) |
| Topic 4 (XR founder's remark on holocaust) | | −0.123** | | −0.158*** | | −0.125** |
| | | (0.043) | | (0.043) | | (0.041) |
| Topic 5 (Disruptive engagement) | | 0.007 | | 0.029 | | 0.099* |
| | | (0.042) | | (0.042) | | (0.041) |
| Topic 6 (Anti London XR protest messages) | | −0.007 | | 0.014 | | 0.014 |
| | | (0.042) | | (0.042) | | (0.040) |
| Topic 7 (Politicized activism ) | | 0.154*** | | 0.200*** | | 0.320*** |
| | | (0.043) | | (0.043) | | (0.042) |
| Burstiness | | −0.010* | | −0.019** | | −0.021*** |
| | | (0.005) | | (0.006) | | (0.006) |
| Observations | 10,547 | 9,300 | 8,687 | 7,680 | 6,827 | 6,000 |
| $R^2$ | 0.007 | 0.044 | 0.006 | 0.057 | 0.007 | 0.128 |
| Adjusted $R^2$ | 0.006 | 0.042 | 0.005 | 0.055 | 0.007 | 0.126 |
| *Note:* | | | | | | *p<0.05; **p<0.01; ***p<0.001 |

**Table 26** Negative Binomial DiD Model Predicting the Average Daily Tweet Count 30 days after interaction with astroturfer bots

| | *Dependent variable:* | | | | | |
|---|---|---|---|---|---|---|
| | Amount | | | | | |
| | 65 | 65 | 70 | 70 | 75 | 75 |
| Constant | 0.626*** | −0.436* | 0.735*** | −0.211 | 0.744*** | 0.441 |
| | (0.098) | (0.194) | (0.100) | (0.239) | (0.138) | (0.360) |
| Bot interaction (yes = 1) | −0.953*** | −0.617*** | −1.209*** | −0.718*** | −1.412*** | −0.943*** |
| | (0.117) | (0.112) | (0.118) | (0.119) | (0.158) | (0.159) |
| After (yes = 1) | −0.254 | −0.234 | −0.247 | −0.243 | −0.072 | −0.209 |
| | (0.140) | (0.131) | (0.142) | (0.141) | (0.195) | (0.188) |
| Bot interaction*After | 0.651*** | 0.455** | 0.339* | 0.393* | 0.076 | 0.159 |
| | (0.165) | (0.156) | (0.168) | (0.167) | (0.224) | (0.214) |
| Sentiment of interaction | | 0.237** | | 0.262** | | 0.319*** |
| | | (0.081) | | (0.089) | | (0.096) |
| Number of retweet of interaction | | −0.0002 | | −0.0002 | | 0.001 |
| | | (0.0004) | | (0.0004) | | (0.0005) |
| Number of likes of interaction | | 0.133*** | | 0.167*** | | 0.097** |
| | | (0.012) | | (0.024) | | (0.032) |
| Topic 1 (Football game protest) | | 0.185 | | 0.041 | | −0.213 |
| | | (0.195) | | (0.238) | | (0.350) |
| Topic 2 (COP25 protest) | | 1.087*** | | 0.947*** | | 1.023** |
| | | (0.209) | | (0.245) | | (0.352) |
| Topic 3 (Anti-XR messages) | | −0.590** | | −1.172*** | | −2.618*** |
| | | (0.212) | | (0.251) | | (0.373) |
| Topic 4 (XR founder's remark on holocaust) | | 0.414* | | 0.122 | | −1.280*** |
| | | (0.202) | | (0.236) | | (0.354) |
| Topic 5 (Disruptive engagement) | | 0.399* | | 0.355 | | −1.206*** |
| | | (0.194) | | (0.234) | | (0.340) |
| Topic 6 (Anti London XR protest messages) | | 0.480* | | 0.416 | | 0.272 |
| | | (0.211) | | (0.249) | | (0.346) |
| Topic 7 (Politicized activism ) | | 0.212 | | 0.114 | | 0.164 |
| | | (0.206) | | (0.241) | | (0.340) |
| Burstiness | | −0.657*** | | −0.644*** | | −0.208*** |
| | | (0.047) | | (0.049) | | (0.056) |
| Observations | 7,051 | 6,660 | 6,391 | 6,000 | 3,931 | 3,660 |
| Log Likelihood | −7,524.297 | −6,852.443 | −6,532.538 | −5,988.802 | −3,761.728 | −3,346.734 |
| Akaike Inf. Crit. | 15,056.590 | 13,734.890 | 13,073.080 | 12,007.600 | 7,531.455 | 6,723.467 |
| *Note:* | | | | | | *p<0.05; **p<0.01; ***p<0.001 |

**Table 27** Linear DiD Model Predicting the Average Sentiment of Tweets 30 days after interaction with astroturfer bots

| | *Dependent variable:* | | | | | |
|---|---|---|---|---|---|---|
| | Sentiment | | | | | |
| | 65 | 65 | 70 | 70 | 75 | 75 |
| Constant | $-0.170^{***}$ | $-0.171^{***}$ | $-0.178^{***}$ | $-0.121^{***}$ | $-0.182^{***}$ | $0.188^{**}$ |
| | (0.014) | (0.028) | (0.015) | (0.034) | (0.022) | (0.058) |
| Bot interaction (yes = 1) | $0.133^{***}$ | $0.097^{***}$ | $0.139^{***}$ | $0.107^{***}$ | $0.126^{***}$ | 0.017 |
| | (0.016) | (0.016) | (0.017) | (0.017) | (0.024) | (0.025) |
| After (yes = 1) | $0.109^{***}$ | $0.105^{***}$ | $0.120^{***}$ | $0.115^{***}$ | $0.147^{***}$ | $0.139^{***}$ |
| | (0.019) | (0.019) | (0.021) | (0.021) | (0.031) | (0.030) |
| Bot interaction*After | $-0.113^{***}$ | $-0.107^{***}$ | $-0.116^{***}$ | $-0.109^{***}$ | $-0.140^{***}$ | $-0.122^{***}$ |
| | (0.023) | (0.023) | (0.024) | (0.024) | (0.034) | (0.034) |
| Sentiment of interaction | | −0.0004 | | $0.035^{**}$ | | 0.0003 |
| | | (0.012) | | (0.013) | | (0.015) |
| Number of retweet of interaction | | 0.0001 | | 0.0001 | | $-0.0003^{***}$ |
| | | (0.0001) | | (0.0001) | | (0.0001) |
| Number of likes of interaction | | $-0.004^{*}$ | | $-0.021^{***}$ | | 0.005 |
| | | (0.002) | | (0.004) | | (0.005) |
| Topic 1 (Football game protest) | | 0.030 | | −0.013 | | $-0.382^{***}$ |
| | | (0.028) | | (0.034) | | (0.057) |
| Topic 2 (COP25 protest) | | $-0.065^{*}$ | | $-0.089^{*}$ | | $-0.403^{***}$ |
| | | (0.030) | | (0.035) | | (0.058) |
| Topic 3 (Anti-XR messages) | | 0.001 | | $-0.090^{**}$ | | $-0.478^{***}$ |
| | | (0.030) | | (0.034) | | (0.055) |
| Topic 4 (XR founder's remark on holocaust) | | $0.100^{***}$ | | $0.086^{**}$ | | $-0.156^{**}$ |
| | | (0.029) | | (0.033) | | (0.056) |
| Topic 5 (Disruptive engagement) | | $-0.061^{*}$ | | $-0.116^{***}$ | | $-0.396^{***}$ |
| | | (0.028) | | (0.033) | | (0.054) |
| Topic 6 (Anti London XR protest messages) | | $0.150^{***}$ | | $0.098^{**}$ | | −0.084 |
| | | (0.031) | | (0.035) | | (0.056) |
| Topic 7 (Politicized activism ) | | $0.109^{***}$ | | 0.061 | | $-0.257^{***}$ |
| | | (0.030) | | (0.034) | | (0.055) |
| Burstiness | | $0.051^{***}$ | | $0.047^{***}$ | | $0.036^{***}$ |
| | | (0.006) | | (0.006) | | (0.008) |
| Observations | 7,051 | 6,660 | 6,391 | 6,000 | 3,931 | 3,660 |
| $R^2$ | 0.011 | 0.047 | 0.012 | 0.055 | 0.009 | 0.103 |
| Adjusted $R^2$ | 0.010 | 0.045 | 0.012 | 0.053 | 0.008 | 0.100 |
| *Note:* | | | | | | $^{*}$p<0.05; $^{**}$p<0.01; $^{***}$p<0.001 |

**Table 28** Negative Binomial DiD Model Predicting the Average Daily Tweet Count 30 days after interaction for users who Support, Neutral or Against XR, botometer CAP=.65

| | *Dependent variable:* | | | | | |
|---|---|---|---|---|---|---|
| | Amount | | | | | |
| | Pro | Pro | Neutral | Neutral | Con | Con |
| | (1) | (2) | (3) | (4) | (5) | (6) |
| Constant | 0.248*** | −0.822** | −2.338*** | −3.156*** | −1.373*** | 0.330 |
| | (0.056) | (0.258) | (0.196) | (0.578) | (0.154) | (0.256) |
| Bot interaction (yes = 1) | −0.561*** | −0.675*** | 0.995*** | 0.693** | 0.985*** | 1.907*** |
| | (0.077) | (0.084) | (0.230) | (0.231) | (0.171) | (0.187) |
| After (yes = 1) | −0.086 | −0.012 | 0.182 | 0.033 | −0.342 | −0.452* |
| | (0.079) | (0.080) | (0.271) | (0.266) | (0.226) | (0.214) |
| Bot interaction*after | 0.494*** | 0.255* | 0.397 | 0.263 | −0.031 | 0.168 |
| | (0.108) | (0.111) | (0.318) | (0.310) | (0.249) | (0.234) |
| Sentiment of interaction | | −0.412*** | | −0.794*** | | 0.352** |
| | | (0.057) | | (0.172) | | (0.125) |
| Opinion of user interaction | | 0.242*** | | 0.439* | | 0.831*** |
| | | (0.060) | | (0.195) | | (0.125) |
| Number of retweet of interaction | | 0.003*** | | 0.0004 | | −0.0003 |
| | | (0.0003) | | (0.001) | | (0.0003) |
| Number of likes of interaction | | −0.015*** | | 0.089** | | 0.0005 |
| | | (0.005) | | (0.030) | | (0.015) |
| Bot score (astroturf) | | 0.279*** | | 0.049 | | −0.176*** |
| | | (0.027) | | (0.068) | | (0.044) |
| Topic 1 (Football game protest) | | 0.018 | | 0.606 | | −0.910*** |
| | | (0.257) | | (0.546) | | (0.233) |
| Topic 2 (COP25 protest) | | 0.854*** | | 0.662 | | −2.258*** |
| | | (0.257) | | (0.514) | | (0.255) |
| Topic 3 (Anti-XR messages) | | −0.285 | | 1.560** | | −3.164*** |
| | | (0.267) | | (0.504) | | (0.387) |
| Topic 4 (XR founder's remark on holocaust) | | 0.930*** | | −0.440 | | −1.899*** |
| | | (0.257) | | (0.541) | | (0.256) |
| Topic 5 (Disruptive engagement) | | 0.584* | | 0.927 | | −2.792*** |
| | | (0.252) | | (0.534) | | (0.264) |
| Topic 6 (Anti London XR protest messages) | | 0.245 | | −0.926 | | −2.504*** |
| | | (0.253) | | (0.613) | | (0.279) |
| Topic 7 (Politicized activism ) | | −0.435 | | 0.708 | | −3.410*** |
| | | (0.259) | | (0.556) | | (0.296) |
| Burstiness | | 0.060 | | 0.177* | | −0.059 |
| | | (0.031) | | (0.076) | | (0.078) |
| Observations | 10,398 | 9,420 | 3,240 | 2,880 | 3,960 | 3,660 |
| Log Likelihood | −11,905.070 | −10,472.790 | −1,654.172 | −1,520.042 | −3,138.700 | −2,779.994 |
| Akaike Inf. Crit. | 23,818.150 | 20,979.580 | 3,316.344 | 3,074.083 | 6,285.401 | 5,593.989 |
| *Note:* | | | | | | *p<0.05; **p<0.01; ***p<0.001 |

**Table 29** Linear DiD Model Predicting the Average Sentiment of Tweets 30 days after interaction for users who Support, Neutral or Against XR, botometer CAP=.65

| | Sentiment | | | | | |
|---|---|---|---|---|---|---|
| | Pro | Pro | Neutral | Neutral | Con | Con |
| | (1) | (2) | (3) | (4) | (5) | (6) |
| Constant | $-0.142^{***}$ | $-0.032$ | $-0.041^{*}$ | $-0.032$ | $-0.108^{***}$ | 0.064 |
| | (0.009) | (0.065) | (0.019) | (0.065) | (0.021) | (0.041) |
| Bot interaction (yes = 1) | $0.148^{***}$ | $-0.007$ | $-0.003$ | $-0.007$ | $0.050^{*}$ | $0.051^{*}$ |
| | (0.012) | (0.024) | (0.023) | (0.024) | (0.023) | (0.024) |
| After (yes = 1) | $0.082^{***}$ | $0.287^{***}$ | $0.267^{***}$ | $0.287^{***}$ | $-0.054$ | $-0.054^{*}$ |
| | (0.013) | (0.027) | (0.026) | (0.027) | (0.029) | (0.026) |
| Bot interaction*after | $-0.110^{***}$ | $-0.248^{***}$ | $-0.236^{***}$ | $-0.248^{***}$ | 0.027 | 0.036 |
| | (0.017) | (0.033) | (0.033) | (0.033) | (0.033) | (0.030) |
| Sentiment of interaction | | $-0.102^{***}$ | | $-0.102^{***}$ | | $0.285^{***}$ |
| | | (0.020) | | (0.020) | | (0.018) |
| Opinion of user interaction | | $0.157^{***}$ | | $0.157^{***}$ | | $-0.042^{*}$ |
| | | (0.019) | | (0.019) | | (0.020) |
| Number of retweet of interaction | | $-0.0004^{***}$ | | $-0.0004^{***}$ | | $-0.0001$ |
| | | (0.0001) | | (0.0001) | | (0.0001) |
| Number of likes of interaction | | $-0.012^{***}$ | | $-0.012^{***}$ | | $-0.014^{***}$ |
| | | (0.003) | | (0.003) | | (0.002) |
| Bot score (astroturf) | | $-0.053^{***}$ | | $-0.053^{***}$ | | $-0.020^{**}$ |
| | | (0.008) | | (0.008) | | (0.007) |
| Topic 1 (Football game protest) | | 0.037 | | 0.037 | | $-0.025$ |
| | | (0.062) | | (0.062) | | (0.037) |
| Topic 2 (COP25 protest) | | $0.137^{*}$ | | $0.137^{*}$ | | $-0.097^{*}$ |
| | | (0.058) | | (0.058) | | (0.038) |
| Topic 3 (Anti-XR messages) | | $0.135^{*}$ | | $0.135^{*}$ | | $-0.064$ |
| | | (0.057) | | (0.057) | | (0.048) |
| Topic 4 (XR founder's remark on holocaust) | | $0.142^{*}$ | | $0.142^{*}$ | | $-0.157^{***}$ |
| | | (0.059) | | (0.059) | | (0.038) |
| Topic 5 (Disruptive engagement) | | 0.041 | | 0.041 | | $-0.255^{***}$ |
| | | (0.060) | | (0.060) | | (0.037) |
| Topic 6 (Anti London XR protest messages) | | $0.229^{***}$ | | $0.229^{***}$ | | $-0.286^{***}$ |
| | | (0.062) | | (0.062) | | (0.041) |
| Topic 7 (Politicized activism ) | | $0.271^{***}$ | | $0.271^{***}$ | | 0.013 |
| | | (0.062) | | (0.062) | | (0.041) |
| Burstiness | | 0.013 | | 0.013 | | 0.012 |
| | | (0.009) | | (0.009) | | (0.011) |
| Observations | 10,398 | 2,880 | 3,240 | 2,880 | 3,960 | 3,660 |
| $R^2$ | 0.016 | 0.142 | 0.048 | 0.142 | 0.005 | 0.115 |
| Adjusted $R^2$ | 0.015 | 0.137 | 0.047 | 0.137 | 0.005 | 0.111 |

*Note:* $^{*}p<0.05$; $^{**}p<0.01$; $^{***}p<0.001$

**Table 30** Negative Binomial DiD Model Predicting the Average Daily Tweet Count 30 days after interaction for users who Support, Neutral or Against XR, botometer CAP=.70

| | *Dependent variable:* | | | | | |
|---|---|---|---|---|---|---|
| | Amount | | | | | |
| | Pro | Pro | Neutral | Neutral | Con | Con |
| | (1) | (2) | (3) | (4) | (5) | (6) |
| Constant | 0.219*** | −3.511*** | −2.303*** | −3.511*** | −1.455*** | −0.203 |
| | (0.060) | (0.531) | (0.197) | (0.531) | (0.170) | (0.264) |
| Bot interaction (yes = 1) | −0.644*** | 0.944*** | 0.588* | 0.944*** | 0.856*** | 1.326*** |
| | (0.083) | (0.234) | (0.231) | (0.234) | (0.185) | (0.206) |
| After (yes = 1) | −0.065 | 0.120 | 0.236 | 0.120 | −0.241 | −0.407 |
| | (0.085) | (0.259) | (0.271) | (0.259) | (0.247) | (0.233) |
| Bot interaction*After | 0.190 | −0.290 | −0.274 | −0.290 | −0.067 | 0.156 |
| | (0.118) | (0.309) | (0.320) | (0.309) | (0.269) | (0.252) |
| Sentiment of interaction | | −1.324*** | | −1.324*** | | 0.031 |
| | | (0.205) | | (0.205) | | (0.130) |
| Opinion of user interaction | | 0.121 | | 0.121 | | 0.323* |
| | | (0.230) | | (0.230) | | (0.126) |
| Number of retweet of interaction | | 0.0004 | | 0.0004 | | −0.0001 |
| | | (0.001) | | (0.001) | | (0.0004) |
| Number of likes of interaction | | 0.0001 | | 0.0001 | | 0.021 |
| | | (0.034) | | (0.034) | | (0.015) |
| Bot score (astroturf) | | 0.200** | | 0.200** | | 0.014 |
| | | (0.075) | | (0.075) | | (0.045) |
| Topic 1 (Football game protest) | | 0.651 | | 0.651 | | −0.585* |
| | | (0.510) | | (0.510) | | (0.259) |
| Topic 2 (COP25 protest) | | 0.416 | | 0.416 | | −1.896*** |
| | | (0.447) | | (0.447) | | (0.286) |
| Topic 3 (Anti-XR messages) | | 0.584 | | 0.584 | | −2.312*** |
| | | (0.455) | | (0.455) | | (0.393) |
| Topic 4 (XR founder's remark on holocaust) | | −0.503 | | −0.503 | | −2.762*** |
| | | (0.483) | | (0.483) | | (0.294) |
| Topic 5 (Disruptive engagement) | | 1.274** | | 1.274** | | −2.482*** |
| | | (0.473) | | (0.473) | | (0.285) |
| Topic 6 (Anti London XR protest messages) | | −0.747 | | −0.747 | | −2.283*** |
| | | (0.592) | | (0.592) | | (0.305) |
| Topic 7 (Politicized activism ) | | 0.857 | | 0.857 | | −2.820*** |
| | | (0.506) | | (0.506) | | (0.312) |
| Burstiness | | 0.019 | | 0.019 | | −0.087 |
| | | (0.085) | | (0.085) | | (0.082) |
| Observations | 8,778 | 2,460 | 2,700 | 2,460 | 3,600 | 3,300 |
| Log Likelihood | −9,543.139 | −1,040.597 | −1,134.655 | −1,040.597 | −2,713.683 | −2,364.884 |
| Akaike Inf. Crit. | 19,094.280 | 2,115.195 | 2,277.310 | 2,115.195 | 5,435.366 | 4,763.769 |
| *Note:* | | | | | | *p<0.05; **p<0.01; ***p<0.001 |

**Table 31** Linear DiD Model Predicting the Average Sentiment of Tweets 30 days after interaction for users who Support, Neutral or Against XR, botometer CAP=.70

| | *Dependent variable:* | | | | | |
|---|---|---|---|---|---|---|
| | Sentiment | | | | | |
| | Pro | Pro | Neutral | Neutral | Con | Con |
| | (1) | (2) | (3) | (4) | (5) | (6) |
| Constant | $-0.147^{***}$ | 0.028 | $-0.063^{**}$ | 0.028 | $-0.134^{***}$ | 0.063 |
| | (0.010) | (0.068) | (0.021) | (0.068) | (0.023) | (0.046) |
| Bot interaction (yes = 1) | $0.141^{***}$ | 0.010 | 0.004 | 0.010 | $0.061^{*}$ | 0.044 |
| | (0.013) | (0.027) | (0.026) | (0.027) | (0.026) | (0.028) |
| After (yes = 1) | $0.075^{***}$ | $0.328^{***}$ | $0.306^{***}$ | $0.328^{***}$ | $-0.031$ | $-0.031$ |
| | (0.014) | (0.029) | (0.030) | (0.029) | (0.033) | (0.030) |
| Bot interaction*After | $-0.077^{***}$ | $-0.279^{***}$ | $-0.267^{***}$ | $-0.279^{***}$ | 0.009 | 0.020 |
| | (0.018) | (0.036) | (0.036) | (0.036) | (0.037) | (0.034) |
| Sentiment of interaction | | $-0.081^{***}$ | | $-0.081^{***}$ | | $0.267^{***}$ |
| | | (0.024) | | (0.024) | | (0.020) |
| Opinion of user interaction | | $0.155^{***}$ | | $0.155^{***}$ | | $-0.044^{*}$ |
| | | (0.021) | | (0.021) | | (0.021) |
| Number of retweet of interaction | | $-0.0004^{***}$ | | $-0.0004^{***}$ | | $-0.0001^{*}$ |
| | | (0.0001) | | (0.0001) | | (0.0001) |
| Number of likes of interaction | | $-0.020^{***}$ | | $-0.020^{***}$ | | $-0.011^{***}$ |
| | | (0.003) | | (0.003) | | (0.002) |
| Bot score (astroturf) | | $-0.073^{***}$ | | $-0.073^{***}$ | | $-0.013$ |
| | | (0.010) | | (0.010) | | (0.007) |
| Topic 1 (Football game protest) | | $0.151^{*}$ | | $0.151^{*}$ | | $-0.080$ |
| | | (0.066) | | (0.066) | | (0.045) |
| Topic 2 (COP25 protest) | | $0.133^{*}$ | | $0.133^{*}$ | | $-0.133^{**}$ |
| | | (0.060) | | (0.060) | | (0.047) |
| Topic 3 (Anti-XR messages) | | 0.077 | | 0.077 | | $-0.172^{**}$ |
| | | (0.060) | | (0.060) | | (0.056) |
| Topic 4 (XR founder's remark on holocaust) | | $0.137^{*}$ | | $0.137^{*}$ | | $-0.197^{***}$ |
| | | (0.060) | | (0.060) | | (0.045) |
| Topic 5 (Disruptive engagement) | | 0.018 | | 0.018 | | $-0.271^{***}$ |
| | | (0.062) | | (0.062) | | (0.045) |
| Topic 6 (Anti London XR protest messages) | | $0.210^{**}$ | | $0.210^{**}$ | | $-0.307^{***}$ |
| | | (0.064) | | (0.064) | | (0.049) |
| Topic 7 (Politicized activism ) | | $0.210^{**}$ | | $0.210^{**}$ | | $-0.009$ |
| | | (0.066) | | (0.066) | | (0.048) |
| Burstiness | | 0.007 | | 0.007 | | $0.038^{**}$ |
| | | (0.010) | | (0.010) | | (0.013) |
| Observations | 8,778 | 2,460 | 2,700 | 2,460 | 3,600 | 3,300 |
| $R^2$ | 0.017 | 0.169 | 0.057 | 0.169 | 0.004 | 0.098 |
| Adjusted $R^2$ | 0.017 | 0.164 | 0.056 | 0.164 | 0.004 | 0.094 |
| *Note:* | | | | | | $^{*}$p<0.05; $^{**}$p<0.01; $^{***}$p<0.001 |

**Table 32** Negative Binomial DiD Model Predicting the Average Daily Tweet Count 30 days after interaction for users who Support, Neutral or Against XR, botometer CAP=.75

| | *Dependent variable:* | | | | | |
|---|---|---|---|---|---|---|
| | Amount | | | | | |
| | Pro | Pro | Neutral | Neutral | Con | Con |
| | (1) | (2) | (3) | (4) | (5) | (6) |
| Constant | 0.060 | −3.378*** | −2.568*** | −3.378*** | −1.415*** | 0.334 |
| | (0.069) | (0.482) | (0.282) | (0.482) | (0.206) | (0.297) |
| Bot interaction (yes = 1) | −0.487*** | 0.716* | 0.758* | 0.716* | 0.306 | 1.256*** |
| | (0.097) | (0.319) | (0.324) | (0.319) | (0.223) | (0.279) |
| After (yes = 1) | 0.082 | 0.084 | 0.197 | 0.084 | −0.061 | −0.153 |
| | (0.097) | (0.365) | (0.388) | (0.365) | (0.294) | (0.292) |
| Bot interaction*After | −0.097 | −0.486 | −0.294 | −0.486 | −0.055 | −0.020 |
| | (0.138) | (0.427) | (0.451) | (0.427) | (0.317) | (0.311) |
| Sentiment of interaction | | −1.588*** | | −1.588*** | | 0.710*** |
| | | (0.297) | | (0.297) | | (0.171) |
| Opinion of user interaction | | 0.518 | | 0.518 | | 0.017 |
| | | (0.266) | | (0.266) | | (0.183) |
| Number of retweet of interaction | | −0.004 | | −0.004 | | −0.001* |
| | | (0.002) | | (0.002) | | (0.0004) |
| Number of likes of interaction | | −0.013 | | −0.013 | | 0.003 |
| | | (0.039) | | (0.039) | | (0.017) |
| Bot score (astroturf) | | 0.252* | | 0.252* | | 0.104 |
| | | (0.102) | | (0.102) | | (0.054) |
| Topic 1 (Football game protest) | | | | | | −1.866*** |
| | | | | | | (0.325) |
| Topic 2 (COP25 protest) | | 0.354 | | 0.354 | | −2.300*** |
| | | (0.446) | | (0.446) | | (0.345) |
| Topic 3 (Anti-XR messages) | | 0.886* | | 0.886* | | −3.175*** |
| | | (0.421) | | (0.421) | | (0.411) |
| Topic 4 (XR founder's remark on holocaust) | | −0.637 | | −0.637 | | −3.689*** |
| | | (0.492) | | (0.492) | | (0.363) |
| Topic 5 (Disruptive engagement) | | 1.694*** | | 1.694*** | | −3.877*** |
| | | (0.451) | | (0.451) | | (0.365) |
| Topic 6 (Anti London XR protest messages) | | −0.329 | | −0.329 | | −3.233*** |
| | | (0.519) | | (0.519) | | (0.386) |
| Topic 7 (Politicized activism ) | | 0.668 | | 0.668 | | −3.562*** |
| | | (0.521) | | (0.521) | | (0.368) |
| Burstiness | | 0.167 | | 0.167 | | −0.014 |
| | | (0.116) | | (0.116) | | (0.104) |
| Observations | 6,198 | 1,680 | 1,920 | 1,680 | 2,640 | 2,340 |
| Log Likelihood | −6,622.960 | −617.740 | −699.774 | −617.740 | −1,740.707 | −1,450.346 |
| Akaike Inf. Crit. | 13,253.920 | 1,267.480 | 1,407.548 | 1,267.480 | 3,489.414 | 2,934.692 |

*Note:* *p<0.05; **p<0.01; ***p<0.001

**Table 33** Linear DiD Model Predicting the Average Sentiment of Tweets 30 days after interaction for users who Support, Neutral or Against XR, botometer CAP=.75

| | *Dependent variable:* | | | | | |
|---|---|---|---|---|---|---|
| | Sentiment | | | | | |
| | Pro | Pro | Neutral | Neutral | Con | Con |
| | (1) | (2) | (3) | (4) | (5) | (6) |
| Constant | $-0.133^{***}$ | $-0.204^{***}$ | 0.010 | $-0.204^{***}$ | $-0.162^{***}$ | $0.234^{***}$ |
| | (0.011) | (0.052) | (0.026) | (0.052) | (0.031) | (0.061) |
| Bot interaction (yes = 1) | $0.098^{***}$ | $-0.002$ | $-0.073^{*}$ | $-0.002$ | $0.081^{*}$ | 0.063 |
| | (0.015) | (0.030) | (0.031) | (0.030) | (0.034) | (0.036) |
| After (yes = 1) | $0.076^{***}$ | $0.356^{***}$ | $0.320^{***}$ | $0.356^{***}$ | $0.099^{*}$ | $0.099^{*}$ |
| | (0.016) | (0.033) | (0.036) | (0.033) | (0.044) | (0.039) |
| Bot interaction*After | $-0.040$ | $-0.320^{***}$ | $-0.297^{***}$ | $-0.320^{***}$ | $-0.114^{*}$ | $-0.099^{*}$ |
| | (0.022) | (0.041) | (0.044) | (0.041) | (0.048) | (0.043) |
| Sentiment of interaction | | $-0.388^{***}$ | | $-0.388^{***}$ | | $0.273^{***}$ |
| | | (0.031) | | (0.031) | | (0.026) |
| Opinion of user interaction | | $0.276^{***}$ | | $0.276^{***}$ | | $-0.130^{***}$ |
| | | (0.022) | | (0.022) | | (0.030) |
| Number of retweet of interaction | | $0.001^{**}$ | | $0.001^{**}$ | | $-0.0003^{***}$ |
| | | (0.0002) | | (0.0002) | | (0.0001) |
| Number of likes of interaction | | $-0.033^{***}$ | | $-0.033^{***}$ | | $-0.012^{***}$ |
| | | (0.004) | | (0.004) | | (0.003) |
| Bot score (astroturf) | | $-0.016$ | | $-0.016$ | | $-0.031^{***}$ |
| | | (0.011) | | (0.011) | | (0.008) |
| Topic 1 (Football game protest) | | | | | | $-0.291^{***}$ |
| | | | | | | (0.063) |
| Topic 2 (COP25 protest) | | 0.013 | | 0.013 | | $-0.367^{***}$ |
| | | (0.050) | | (0.050) | | (0.065) |
| Topic 3 (Anti-XR messages) | | $0.351^{***}$ | | $0.351^{***}$ | | $-0.412^{***}$ |
| | | (0.048) | | (0.048) | | (0.069) |
| Topic 4 (XR founder's remark on holocaust) | | $0.152^{**}$ | | $0.152^{**}$ | | $-0.355^{***}$ |
| | | (0.047) | | (0.047) | | (0.063) |
| Topic 5 (Disruptive engagement) | | $0.315^{***}$ | | $0.315^{***}$ | | $-0.456^{***}$ |
| | | (0.051) | | (0.051) | | (0.065) |
| Topic 6 (Anti London XR protest messages) | | $0.479^{***}$ | | $0.479^{***}$ | | $-0.527^{***}$ |
| | | (0.049) | | (0.049) | | (0.068) |
| Topic 7 (Politicized activism ) | | $0.546^{***}$ | | $0.546^{***}$ | | $-0.159^{*}$ |
| | | (0.054) | | (0.054) | | (0.064) |
| Burstiness | | $-0.018$ | | $-0.018$ | | $0.085^{***}$ |
| | | (0.011) | | (0.011) | | (0.017) |
| Observations | 6,198 | 1,680 | 1,920 | 1,680 | 2,640 | 2,340 |
| $R^2$ | 0.013 | 0.307 | 0.087 | 0.307 | 0.002 | 0.122 |
| Adjusted $R^2$ | 0.012 | 0.300 | 0.086 | 0.300 | 0.001 | 0.116 |
| *Note:* | | | | | | $^{*}$p<0.05; $^{**}$p<0.01; $^{***}$p<0.001 |

**Table 34** Linear DiD Model Predicting the Change in Support 30 days after interaction for users who Support, Neutral or Against XR, with varied botometer CAP scores, without controls

| | *Dependent variable:* | | | | | | | | |
|---|---|---|---|---|---|---|---|---|---|
| | Opinion Change | | | | | | | | |
| | Pro | Neutral | Con | Pro | Neutral | Con | Pro | Neutral | Con |
| Constant | $0.172^{***}$ | $0.105^{***}$ | $-0.520^{***}$ | $0.204^{***}$ | $0.133^{***}$ | $-0.583^{***}$ | $0.204^{***}$ | $0.133^{***}$ | $-0.857^{***}$ |
| | (0.009) | (0.014) | (0.026) | (0.010) | (0.016) | (0.029) | (0.010) | (0.016) | (0.037) |
| Bot interaction (yes = 1) | −0.008 | $-0.220^{***}$ | $0.195^{***}$ | $-0.051^{***}$ | $-0.217^{***}$ | $0.265^{***}$ | $-0.051^{***}$ | $-0.217^{***}$ | $0.525^{***}$ |
| | (0.012) | (0.018) | (0.030) | (0.013) | (0.020) | (0.032) | (0.013) | (0.020) | (0.041) |
| After (yes = 1) | −0.001 | −0.000 | −0.000 | −0.001 | 0.000 | −0.000 | −0.001 | 0.000 | −0.000 |
| | (0.013) | (0.020) | (0.037) | (0.014) | (0.023) | (0.040) | (0.014) | (0.023) | (0.053) |
| Bot interaction*After | −0.003 | 0.000 | 0.000 | −0.004 | −0.000 | 0.000 | −0.004 | −0.000 | 0.000 |
| | (0.017) | (0.025) | (0.042) | (0.019) | (0.028) | (0.045) | (0.019) | (0.028) | (0.058) |
| CAP | 65 | 70 | 75 | 65 | 70 | 75 | 65 | 70 | 75 |
| Observations | 10,398 | 3,240 | 3,960 | 8,778 | 2,700 | 3,600 | 8,778 | 2,700 | 2,640 |
| $R^2$ | 0.0001 | 0.086 | 0.021 | 0.004 | 0.082 | 0.037 | 0.004 | 0.082 | 0.112 |
| Adjusted $R^2$ | −0.0002 | 0.085 | 0.021 | 0.003 | 0.081 | 0.036 | 0.003 | 0.081 | 0.111 |
| *Note:* | | | | | | | $^{*}p<0.05$; $^{**}p<0.01$; $^{***}p<0.001$ | | |

**Table 35** Linear DiD Model Predicting the Change in Support 30 days after interaction for users who Support, Neutral or Against XR, with varied botometer CAP scores, with controls

| | *Dependent variable:* | | | | | | | | |
|---|---|---|---|---|---|---|---|---|---|
| | Opinion Change | | | | | | | | |
| | Pro | Neutral | Con | Pro | Neutral | Con | Pro | Neutral | Con |
| Constant | 0.819*** | −0.793*** | −0.133** | 0.868*** | −0.798*** | −0.061 | 0.868*** | −0.798*** | −0.031 |
| | (0.040) | (0.048) | (0.051) | (0.041) | (0.042) | (0.056) | (0.041) | (0.042) | (0.068) |
| Bot interaction (yes = 1) | 0.070*** | −0.214*** | 0.389*** | 0.026 | −0.106*** | 0.490*** | 0.026 | −0.106*** | 0.705*** |
| | (0.013) | (0.018) | (0.030) | (0.014) | (0.016) | (0.034) | (0.014) | (0.016) | (0.039) |
| After (yes = 1) | −0.000 | 0.000 | −0.000 | 0.000 | −0.000 | 0.000 | 0.000 | −0.000 | −0.000 |
| | (0.013) | (0.020) | (0.033) | (0.014) | (0.018) | (0.036) | (0.014) | (0.018) | (0.043) |
| Bot interaction*After | 0.000 | 0.000 | 0.000 | 0.000 | 0.000 | −0.000 | 0.000 | 0.000 | 0.000 |
| | (0.017) | (0.024) | (0.038) | (0.019) | (0.022) | (0.041) | (0.019) | (0.022) | (0.048) |
| Sentiment of interaction | −0.044*** | −0.081*** | −0.328*** | −0.040*** | −0.136*** | −0.214*** | −0.040*** | −0.136*** | −0.067* |
| | (0.009) | (0.015) | (0.022) | (0.010) | (0.015) | (0.024) | (0.010) | (0.015) | (0.028) |
| Opinion of user interaction | −0.027** | −0.073*** | 0.243*** | −0.027** | 0.022 | 0.243*** | −0.027** | 0.022 | −0.250*** |
| | (0.009) | (0.014) | (0.025) | (0.010) | (0.013) | (0.026) | (0.010) | (0.013) | (0.033) |
| Num. of retweet of interaction | −0.0003*** | 0.00003 | −0.001*** | −0.0004*** | 0.0004*** | −0.001*** | −0.0004*** | 0.0004*** | −0.001*** |
| | (0.0001) | (0.0001) | (0.0001) | (0.0001) | (0.0001) | (0.0001) | (0.0001) | (0.0001) | (0.0001) |
| Num. of likes of interaction | 0.008*** | 0.020*** | 0.037*** | 0.007*** | 0.026*** | 0.030*** | 0.007*** | 0.026*** | 0.019*** |
| | (0.001) | (0.002) | (0.003) | (0.001) | (0.002) | (0.003) | (0.001) | (0.002) | (0.003) |
| Bot score (astroturf) | −0.090*** | −0.003 | 0.012 | −0.099*** | −0.052*** | −0.023** | −0.099*** | −0.052*** | −0.027** |
| | (0.004) | (0.006) | (0.008) | (0.005) | (0.006) | (0.009) | (0.005) | (0.006) | (0.009) |
| Topic 1 (Football game protest) | −0.614*** | 1.078*** | −0.385*** | −0.537*** | 1.396*** | −0.417*** | −0.537*** | 1.396*** | −0.662*** |
| | (0.040) | (0.046) | (0.046) | (0.042) | (0.041) | (0.054) | (0.042) | (0.041) | (0.070) |
| Topic 2 (COP25 protest) | −0.518*** | 0.723*** | −0.503*** | −0.540*** | 0.836*** | −0.572*** | −0.540*** | 0.836*** | −1.076*** |
| | (0.040) | (0.043) | (0.047) | (0.041) | (0.036) | (0.057) | (0.041) | (0.036) | (0.072) |
| Topic 3 (Anti-XR messages) | −0.292*** | 0.868*** | −0.602*** | −0.188*** | 0.872*** | −0.748*** | −0.188*** | 0.872*** | −0.921*** |
| | (0.041) | (0.043) | (0.059) | (0.042) | (0.037) | (0.067) | (0.042) | (0.037) | (0.076) |
| Topic 4 (XR founder's remark on holocaust) | −0.562*** | 0.928*** | −0.646*** | −0.552*** | 0.852*** | −0.592*** | −0.552*** | 0.852*** | −0.988*** |
| | (0.040) | (0.044) | (0.047) | (0.041) | (0.037) | (0.055) | (0.041) | (0.037) | (0.069) |
| Topic 5 (Disruptive engagement) | −0.415*** | 0.887*** | −0.665*** | −0.418*** | 0.934*** | −0.745*** | −0.418*** | 0.934*** | −1.368*** |
| | (0.039) | (0.045) | (0.046) | (0.040) | (0.038) | (0.055) | (0.040) | (0.038) | (0.071) |
| Topic 6 (Anti London XR protest messages) | −0.540*** | 0.803*** | −0.504*** | −0.539*** | 0.860*** | −0.583*** | −0.539*** | 0.860*** | −1.180*** |
| | (0.039) | (0.046) | (0.050) | (0.040) | (0.039) | (0.059) | (0.040) | (0.039) | (0.075) |
| Topic 7 (Politicized activism ) | −0.451*** | 0.978*** | −0.621*** | −0.464*** | 0.986*** | −0.675*** | −0.464*** | 0.986*** | −0.787*** |
| | (0.040) | (0.046) | (0.050) | (0.040) | (0.040) | (0.058) | (0.040) | (0.040) | (0.070) |
| Burstiness | 0.005 | 0.025*** | −0.014 | 0.009 | 0.094*** | −0.135*** | 0.009 | 0.094*** | −0.213*** |
| | (0.005) | (0.006) | (0.013) | (0.005) | (0.006) | (0.015) | (0.005) | (0.006) | (0.019) |
| CAP | 65 | 70 | 75 | 65 | 70 | 75 | 65 | 70 | 75 |
| Observations | 9,420 | 2,880 | 3,660 | 7,920 | 2,460 | 3,300 | 7,920 | 2,460 | 2,340 |
| $R^2$ | 0.118 | 0.348 | 0.238 | 0.132 | 0.537 | 0.229 | 0.132 | 0.537 | 0.420 |
| Adjusted $R^2$ | 0.117 | 0.344 | 0.234 | 0.130 | 0.534 | 0.225 | 0.130 | 0.534 | 0.416 |
| *Note:* | | | | | | | | | *p<0.05; **p<0.01; ***p<0.001 |

**Table 36** Negative Binomial DiD Model Predicting the Average Daily Tweet Count 30 days after interaction with astroturfing bots, Coarsened Exact Matching only

| | *Dependent variable:* | | | | | |
|---|---|---|---|---|---|---|
| | Amount | | | | | |
| | 65 | 65 | 70 | 70 | 75 | 75 |
| | (1) | (2) | (3) | (4) | (5) | (6) |
| Bot interaction | −0.064 | −0.346** | −0.380** | −0.465*** | −0.408* | −0.530** |
| | (0.134) | (0.123) | (0.125) | (0.127) | (0.171) | (0.174) |
| After | −0.081 | −0.156 | −0.058 | −0.163 | −0.087 | −0.389 |
| | (0.166) | (0.146) | (0.154) | (0.152) | (0.212) | (0.212) |
| Sentiment of Interaction | | 0.480*** | | 0.748*** | | 0.677*** |
| | | (0.083) | | (0.088) | | (0.103) |
| Number of retweets | | −0.004*** | | −0.008*** | | −0.006** |
| | | (0.001) | | (0.001) | | (0.002) |
| Number of likes | | 0.212*** | | 0.078** | | 0.181*** |
| | | (0.016) | | (0.026) | | (0.032) |
| Topic 1 (Football game protest) | | 0.658** | | 0.351 | | 0.147 |
| | | (0.202) | | (0.216) | | (0.343) |
| Topic 2 (COP25 protest) | | 1.017*** | | 0.660** | | −1.044* |
| | | (0.230) | | (0.238) | | (0.430) |
| Topic 3 (Anti-XR messages) | | −0.011 | | −0.688** | | −1.990*** |
| | | (0.221) | | (0.255) | | (0.417) |
| Topic 4 (XR founder's remark on holocaust) | | −0.311 | | −0.423 | | −0.894* |
| | | (0.212) | | (0.226) | | (0.349) |
| Topic 5 (Disruptive engagement) | | 0.186 | | −0.267 | | −0.929** |
| | | (0.208) | | (0.219) | | (0.346) |
| Topic 6 (Anti London XR protest messages) | | 1.289*** | | 1.004*** | | 0.908** |
| | | (0.213) | | (0.223) | | (0.345) |
| Topic 7 (Politicized activism ) | | 0.313 | | −0.029 | | −0.108 |
| | | (0.214) | | (0.219) | | (0.342) |
| Burstiness | | −0.208*** | | −0.132* | | −0.059 |
| | | (0.049) | | (0.052) | | (0.063) |
| Bot interaction:After | 0.486* | 0.157 | −0.090 | 0.003 | −0.014 | 0.174 |
| | (0.190) | (0.170) | (0.178) | (0.176) | (0.243) | (0.240) |
| Constant | −0.438*** | −1.200*** | −0.374*** | −0.560* | −0.353* | −0.252 |
| | (0.117) | (0.202) | (0.109) | (0.220) | (0.149) | (0.360) |
| Observations | 5,551 | 5,160 | 4,951 | 4,560 | 3,271 | 3,000 |
| Log Likelihood | −5,232.702 | −4,529.911 | −4,206.804 | −3,689.154 | −2,694.084 | −2,331.438 |
| Akaike Inf. Crit. | 10,473.410 | 9,089.822 | 8,421.608 | 7,408.308 | 5,396.168 | 4,692.875 |
| *Note:* | | | | | | *p<0.05; **p<0.01; ***p<0.001 |

**Table 37** Linear DiD Model Predicting the Average Sentiment 30 days after interaction with astroturfing bots, Coarsened Exact Matching only

| [-1.8ex] | *Dependent variable:* | | | | | |
|---|---|---|---|---|---|---|
| | Sentiment | | | | | |
| | 65 | 65 | 70 | 70 | 75 | 75 |
| | (1) | (2) | (3) | (4) | (5) | (6) |
| Bot interaction | 0.135*** | 0.097*** | 0.127*** | 0.086*** | 0.132*** | 0.037 |
| | (0.019) | (0.020) | (0.021) | (0.022) | (0.027) | (0.028) |
| After | 0.120*** | 0.114*** | 0.115*** | 0.107*** | 0.168*** | 0.160*** |
| | (0.024) | (0.024) | (0.026) | (0.026) | (0.033) | (0.033) |
| Sentiment of Interaction | | −0.019 | | 0.021 | | −0.023 |
| | | (0.013) | | (0.014) | | (0.016) |
| Number of retweets | | 0.0004** | | 0.00004 | | −0.0001 |
| | | (0.0001) | | (0.0002) | | (0.0002) |
| Number of likes | | −0.010*** | | −0.030*** | | −0.001 |
| | | (0.003) | | (0.004) | | (0.005) |
| Topic 1 (Football game protest) | | −0.016 | | −0.034 | | −0.399*** |
| | | (0.032) | | (0.036) | | (0.059) |
| Topic 2 (COP25 protest) | | −0.100** | | −0.078* | | −0.435*** |
| | | (0.036) | | (0.040) | | (0.067) |
| Topic 3 (Anti-XR messages) | | −0.017 | | −0.086* | | −0.485*** |
| | | (0.034) | | (0.040) | | (0.063) |
| Topic 4 (XR founder's remark on holocaust) | | 0.085** | | 0.100** | | −0.170** |
| | | (0.032) | | (0.036) | | (0.059) |
| Topic 5 (Disruptive engagement) | | −0.094** | | −0.111** | | −0.416*** |
| | | (0.032) | | (0.036) | | (0.058) |
| Topic 6 (Anti London XR protest messages) | | 0.075* | | 0.041 | | −0.148* |
| | | (0.034) | | (0.038) | | (0.060) |
| Topic 7 (Politicized activism ) | | 0.078* | | 0.059 | | −0.285*** |
| | | (0.033) | | (0.036) | | (0.058) |
| Burstiness | | 0.044*** | | 0.041*** | | 0.032*** |
| | | (0.007) | | (0.008) | | (0.009) |
| Bot interaction:After | −0.130*** | −0.122*** | −0.115*** | −0.105*** | −0.184*** | −0.166*** |
| | (0.027) | (0.028) | (0.030) | (0.030) | (0.038) | (0.037) |
| Constant | −0.167*** | −0.135*** | −0.162*** | −0.091* | −0.176*** | 0.204*** |
| | (0.017) | (0.032) | (0.018) | (0.036) | (0.024) | (0.061) |
| Observations | 5,551 | 5,160 | 4,951 | 4,560 | 3,271 | 3,000 |
| $R^2$ | 0.009 | 0.044 | 0.008 | 0.052 | 0.009 | 0.099 |
| Adjusted $R^2$ | 0.009 | 0.041 | 0.008 | 0.049 | 0.008 | 0.095 |
| *Note:* | | | | | | *p<0.05; **p<0.01; ***p<0.001 |

**Table 38** Negative Binomial DiD Model Predicting the Average Daily Tweet Count 30 days after interaction with bots, with user region controlled

| [-1.8ex] | *Dependent variable:* | | | | | |
|---|---|---|---|---|---|---|
| | Amount | | | | | |
| | 65 | 65 | 70 | 70 | 75 | 75 |
| | (1) | (2) | (3) | (4) | (5) | (6) |
| Constant | −0.056 | 0.141 | −0.188** | 0.602*** | −0.066 | 1.010*** |
| | p = 0.274 | p = 0.358 | p = 0.004 | p = 0.0005 | p = 0.230 | p = 0.00000 |
| Bot interaction | −0.414*** | −0.303*** | −0.608*** | −0.337*** | −0.567*** | −0.266** |
| | p = 0.000 | p = 0.00001 | p = 0.000 | p = 0.00001 | p = 0.000 | p = 0.002 |
| After | −0.090 | −0.124 | 0.078 | −0.115 | −0.066 | −0.061 |
| | p = 0.215 | p = 0.092 | p = 0.395 | p = 0.138 | p = 0.395 | p = 0.498 |
| Bot interaction * After | 0.333*** | 0.237* | −0.126 | 0.045 | 0.059 | 0.028 |
| | p = 0.0004 | p = 0.012 | p = 0.288 | p = 0.659 | p = 0.553 | p = 0.813 |
| Sentiment of Interaction | | −0.090 | | 0.011 | | 0.075 |
| | | p = 0.066 | | p = 0.841 | | p = 0.205 |
| Number of retweets | | 0.001*** | | 0.0003 | | −0.00000 |
| | | p = 0.00000 | | p = 0.230 | | p = 0.988 |
| Number of likes | | −0.003 | | −0.026*** | | −0.031*** |
| | | p = 0.500 | | p = 0.00000 | | p = 0.000 |
| Topic 1 (Football game protest) | | −0.630*** | | −0.776*** | | −1.283*** |
| | | p = 0.00004 | | p = 0.00001 | | p = 0.000 |
| Topic 2 (COP25 protest) | | −0.051 | | −0.524** | | −0.592** |
| | | p = 0.737 | | p = 0.002 | | p = 0.003 |
| Topic 3 (Anti-XR messages) | | −0.463** | | −1.942*** | | −2.493*** |
| | | p = 0.004 | | p = 0.000 | | p = 0.000 |
| Topic 4 (XR founder's remark on holocaust) | | −0.147 | | −0.628*** | | −1.557*** |
| | | p = 0.346 | | p = 0.0003 | | p = 0.000 |
| Topic 5 (Disruptive engagement) | | −0.504*** | | −0.703*** | | −0.829*** |
| | | p = 0.001 | | p = 0.00002 | | p = 0.00002 |
| Topic 6 (Anti London XR protest messages) | | −0.517*** | | −0.894*** | | −1.181*** |
| | | p = 0.001 | | p = 0.00000 | | p = 0.000 |
| Topic 7 (Politicized activism) | | −1.098*** | | −1.323*** | | −1.288*** |
| | | p = 0.000 | | p = 0.000 | | p = 0.000 |
| Australia and NZ | | 1.645*** | | 1.497*** | | 1.128*** |
| | | p = 0.000 | | p = 0.000 | | p = 0.000 |
| Canada | | −0.868*** | | −0.318 | | 0.447 |
| | | p = 0.00001 | | p = 0.085 | | p = 0.122 |
| Europe other | | −0.838*** | | −1.111*** | | −1.181*** |
| | | p = 0.000 | | p = 0.000 | | p = 0.000 |
| Other | | 1.085*** | | −0.462 | | −0.317 |
| | | p = 0.00000 | | p = 0.129 | | p = 0.300 |
| UK and Ireland | | −0.463*** | | −0.469*** | | −0.689*** |
| | | p = 0.000 | | p = 0.000 | | p = 0.000 |
| USA | | 0.448*** | | 0.344*** | | −0.234** |
| | | p = 0.000 | | p = 0.00001 | | p = 0.006 |
| Burstiness | | −0.021 | | −0.203*** | | −0.192*** |
| | | p = 0.454 | | p = 0.000 | | p = 0.00001 |
| Observations | 17,598 | 14,580 | 10,758 | 12,600 | 15,078 | 9,060 |
| Log Likelihood | −17,045.970 | −13,986.710 | −9,297.998 | −11,323.400 | −13,693.760 | −7,967.407 |
| Akaike Inf. Crit. | 34,099.940 | 28,015.410 | 18,604.000 | 22,688.790 | 27,395.530 | 15,976.810 |
| *Note:* | | | | | | *p<0.05; **p<0.01; ***p<0.001 |

**Table 39** Linear DiD Model Predicting the Average Sentiment 30 days after interaction with bots, with demographic controlled

| | *Dependent variable:* | | | | | |
|---|---|---|---|---|---|---|
| | Sentiment | | | | | |
| | 65 | 65 | 70 | 70 | 75 | 75 |
| | (1) | (2) | (3) | (4) | (5) | (6) |
| Constant | −0.121*** | −0.244*** | −0.132*** | −0.249*** | −0.114*** | −0.228*** |
| | p = 0.000 | p = 0.000 | p = 0.000 | p = 0.000 | p = 0.000 | p = 0.000 |
| Bot interaction (yes = 1) | 0.099*** | 0.090*** | 0.095*** | 0.092*** | 0.059*** | 0.056*** |
| | p = 0.000 | p = 0.000 | p = 0.000 | p = 0.000 | p = 0.00001 | p = 0.00003 |
| After | 0.095*** | 0.087*** | 0.098*** | 0.097*** | 0.115*** | 0.119*** |
| | p = 0.000 | p = 0.000 | p = 0.000 | p = 0.000 | p = 0.000 | p = 0.000 |
| Bot interaction: After | −0.112*** | −0.117*** | −0.099*** | −0.113*** | −0.098*** | −0.116*** |
| | p = 0.000 | p = 0.000 | p = 0.000 | p = 0.000 | p = 0.000 | p = 0.000 |
| Sentiment of Interaction | | 0.026*** | | 0.019* | | 0.002 |
| | | p = 0.001 | | p = 0.023 | | p = 0.850 |
| Number of retweets | | 0.0002*** | | 0.0001** | | 0.0002*** |
| | | p = 0.00002 | | p = 0.002 | | p = 0.00000 |
| Number of likes | | −0.002*** | | −0.003*** | | −0.003*** |
| | | p = 0.0005 | | p = 0.0003 | | p = 0.0004 |
| Topic 1 (Football game protest) | | 0.094*** | | 0.027 | | 0.025 |
| | | p = 0.0001 | | p = 0.327 | | p = 0.458 |
| Topic 2 (COP25 protest) | | 0.101*** | | 0.089*** | | 0.008 |
| | | p = 0.00004 | | p = 0.001 | | p = 0.810 |
| Topic 3 (Anti-XR messages) | | 0.148*** | | 0.129*** | | 0.122*** |
| | | p = 0.000 | | p = 0.00001 | | p = 0.0005 |
| Topic 4 (XR founder's remark on holocaust) | | 0.057* | | 0.027 | | −0.004 |
| | | p = 0.023 | | p = 0.322 | | p = 0.902 |
| Topic 5 (Disruptive engagement) | | 0.038 | | 0.041 | | 0.038 |
| | | p = 0.113 | | p = 0.121 | | p = 0.254 |
| Topic 6 (Anti London XR protest messages) | | 0.090*** | | 0.081** | | 0.053 |
| | | p = 0.0002 | | p = 0.002 | | p = 0.103 |
| Topic 7 (Politicized activism ) | | 0.186*** | | 0.188*** | | 0.146*** |
| | | p = 0.000 | | p = 0.000 | | p = 0.00002 |
| Australia and NZ | | −0.022 | | −0.020 | | 0.001 |
| | | p = 0.166 | | p = 0.269 | | p = 0.953 |
| Canada | | −0.015 | | 0.003 | | 0.243*** |
| | | p = 0.585 | | p = 0.912 | | p = 0.00000 |
| Europe other | | 0.133*** | | 0.150*** | | 0.107*** |
| | | p = 0.000 | | p = 0.000 | | p = 0.00001 |
| Other | | −0.049 | | −0.153*** | | −0.123** |
| | | p = 0.150 | | p = 0.0002 | | p = 0.003 |
| UK and Ireland | | 0.084*** | | 0.082*** | | 0.114*** |
| | | p = 0.000 | | p = 0.000 | | p = 0.000 |
| USA | | 0.011 | | 0.023* | | 0.047*** |
| | | p = 0.333 | | p = 0.047 | | p = 0.001 |
| Burstiness | | 0.007 | | 0.004 | | −0.015* |
| | | p = 0.083 | | p = 0.372 | | p = 0.019 |
| Observations | 17,598 | 14,580 | 15,078 | 12,600 | 10,758 | 9,060 |
| $R^2$ | 0.007 | 0.031 | 0.007 | 0.035 | 0.007 | 0.042 |
| Adjusted $R^2$ | 0.007 | 0.029 | 0.007 | 0.034 | 0.006 | 0.040 |
| *Note:* | | | | | | *p<0.05; **p<0.01; ***p<0.001 |

# References


[1] Yang, K., Ferrara, E., Menczer, F.: Botometer 101: Social bot practicum for computational social scientists. CoRR **abs/2201.01608** (2022) 2201.01608

[2] Rauchfleisch, A., Kaiser, J.: The false positive problem of automatic bot detection in social science research. PLOS ONE **15**(10), 1–20 (2020) https://doi.org/10.1371/journal.pone.0241045

[3] Martini, F., Samula, P., Keller, T.R., Klinger, U.: Bot, or not? Comparing three methods for detecting social bots in five political discourses. Big Data & Society **8**(2), 205395172110335 (2021) https://doi.org/10.1177/20539517211033566

[4] González-Bailón, S., De Domenico, M.: Bots are less central than verified accounts during contentious political events. Proceedings of the National Academy of Sciences of the United States of America **118**(11) (2021) https://doi.org/10.1073/pnas.2013443118

[5] Feng, S., Wan, H., Wang, N., Li, J., Luo, M.: Twibot-20: A comprehensive twitter bot detection benchmark. In: Proceedings of the 30th ACM International Conference on Information & Knowledge Management, pp. 4485–4494 (2021)

[6] Feng, S., Tan, Z., Wan, H., Wang, N., Chen, Z., Zhang, B., Zheng, Q., Zhang, W., Lei, Z., Yang, S., Feng, X., Zhang, Q., Wang, H., Liu, Y., Bai, Y., Wang, H., Cai, Z., Wang, Y., Zheng, L., Ma, Z., Li, J., Luo, M.: TwiBot-22: Towards Graph-Based Twitter Bot Detection. Advances in Neural Information Processing Systems, 1–25 (2022) arXiv:2206.04564

[7] Abadi, M., Agarwal, A., Barham, P., Brevdo, E., Chen, Z., Citro, C., Corrado, G.S., Davis, A., Dean, J., Devin, M., Ghemawat, S., Goodfellow, I., Harp, A., Irving, G., Isard, M., Jia, Y., Jozefowicz, R., Kaiser, L., Kudlur, M., Levenberg, J., Mané, D., Monga, R., Moore, S., Murray, D., Olah, C., Schuster, M., Shlens, J., Steiner, B., Sutskever, I., Talwar, K., Tucker, P., Vanhoucke, V., Vasudevan, V., Viégas, F., Vinyals, O., Warden, P., Wattenberg, M., Wicke, M., Yu, Y., Zheng, X.: TensorFlow: Large-Scale Machine Learning on Heterogeneous Systems. Software available from tensorflow.org (2015). https://www.tensorflow.org/

[8] Chollet, F., et al.: Keras. https://keras.io (2015)

[9] Yang, K.-C., Varol, O., Hui, P.-M., Menczer, F.: Scalable and Generalizable Social Bot Detection through Data Selection. Proceedings of the AAAI Conference on Artificial Intelligence **34**(01), 1096–1103 (2020) https://doi.org/10.1609/aaai.v34i01.5460 arXiv:1911.09179

[10] Greve, H.R., Rao, H., Vicinanza, P., Zhou, E.Y.: Online Conspiracy Groups: Micro-Bloggers, Bots, and Coronavirus Conspiracy Talk on Twitter. American Sociological Review (2022) https://doi.org/10.1177/00031224221125937

[11] Yan, X., Guo, J., Lan, Y., Cheng, X.: 2013-a Bitem Topic Model for Short Texts.Pdf. Www, 1445–1455 (2013)

[12] Shi, L., Cheng, G., Xie, S.R., Xie, G.: A word embedding topic model for topic detection and summary in social networks. Measurement and Control (United Kingdom) **52**(9-10), 1289–1298 (2019) https://doi.org/10.1177/0020294019865750

[13] Bird, S., Klein, E., Loper, E.: Natural Language Processing with Python: Analyzing Text with the Natural Language Toolkit. " O'Reilly Media, Inc.", ??? (2009)

[14] Röder, M., Both, A., Hinneburg, A.: Exploring the space of topic coherence measures. In: Proceedings of the Eighth ACM International Conference on Web Search and Data Mining. WSDM '15, pp. 399–408. Association for Computing Machinery, New York, NY, USA (2015). https://doi.org/10.1145/2684822.2685324 . https://doi.org/10.1145/2684822.2685324

[15] Harvard and Yale students disrupt football game for fossil fuel protest — US universities (2019). https://www.theguardian.com/us-news/2019/nov/23/harvard-yale-football-game-protest-fossil-fuels Accessed 2023-02-16

[16] News, B.: COP25: Thousands gather for change climate protests in Madrid — bbc.co.uk. https://www.bbc.co.

uk/news/world-europe-50694361. [Accessed 07-09-2023]

[17] Gayle, D.: Extinction Rebellion bee protester glues himself to Lib Dem bus — theguardian.com. https://www.theguardian.com/environment/2019/dec/04/extinction-rebellion-protester-glues-himself-lib-dem-battlebus. [Accessed 07-09-2023]

[18] Cohen, J.: Weighted kappa: Nominal scale agreement provision for scaled disagreement or partial credit. Psychological bulletin **70**(4), 213 (1968)
